# Active repression of cell fate plasticity by PROX1 safeguards hepatocyte identity and prevents liver tumorigenesis

**DOI:** 10.1038/s41588-025-02081-w

**Published:** 2025-02-13

**Authors:** Bryce Lim, Aryan Kamal, Borja Gomez Ramos, Juan M. Adrian Segarra, Ignacio L. Ibarra, Lennart Dignas, Tim Kindinger, Kai Volz, Mohammad Rahbari, Nuh Rahbari, Eric Poisel, Kanela Kafetzopoulou, Lio Böse, Marco Breinig, Danijela Heide, Suchira Gallage, Jose E. Barragan Avila, Hendrik Wiethoff, Ivan Berest, Sarah Schnabellehner, Martin Schneider, Jonas Becker, Dominic Helm, Dirk Grimm, Taija Mäkinen, Darjus F. Tschaharganeh, Mathias Heikenwalder, Judith B. Zaugg, Moritz Mall

**Affiliations:** 1 Cell Fate Engineering and Disease Modeling Group, German Cancer Research Center (DKFZ) and DKFZ–ZMBH Alliance, Heidelberg, Germany; 2 HITBR Hector Institute for Translational Brain Research gGmbH, Heidelberg, Germany; 3 Central Institute of Mental Health, Medical Faculty Mannheim, Heidelberg University, Mannheim, Germany; 4 Faculty of Biosciences, Heidelberg University, Heidelberg, Germany; 5 European Molecular Biology Laboratory, Molecular Systems Biology Unit, Heidelberg, Germany; 6 Cell Plasticity and Epigenetic Remodeling Helmholtz Group, DKFZ, Heidelberg, Germany; 7 Institute of Pathology, University Hospital, Heidelberg, Germany; 8 Division of Chronic Inflammation and Cancer, DKFZ, Heidelberg, Germany; 9 Department of Surgery, University Hospital Mannheim, Medical Faculty Mannheim, Heidelberg University, Mannheim, Germany; 10 Department of General and Visceral Surgery, University of Ulm, Ulm, Germany; 11 Institute for Interdisciplinary Research on Cancer Metabolism and Chronic Inflammation, M3-Research Center for Malignome, Metabolome and Microbiome, Faculty of Medicine, University Tuebingen, Tübingen, Germany; 12 Department of Immunology, Genetics and Pathology, Uppsala University, Uppsala, Sweden; 13 Proteomics Core Facility, DKFZ, Heidelberg, Germany; 14 Department of Infectious Diseases/Virology, Section Viral Vector Technologies, Medical Faculty and Faculty of Engineering Sciences, Heidelberg University, Center for Integrative Infectious Diseases Research (CIID), BioQuant, Heidelberg, Germany; 15 German Center for Infection Research (DZIF), Partner Site Heidelberg, Heidelberg, Germany; 16 German Center for Cardiovascular Research (DZHK), Partner Site Heidelberg, Heidelberg, Germany; 17 Translational Cancer Medicine Program and Department of Biochemistry and Developmental Biology, University of Helsinki, Helsinki, Finland; 18 Wihuri Research Institute, Helsinki, Finland; 19 Present Address: Department of Biomedicine, University Hospital Basel, University of Basel, Basel, Switzerland

**Keywords:** Liver cancer, Epigenetics, High-throughput screening

## Abstract

Cell fate plasticity enables development, yet unlocked plasticity is a cancer hallmark. While transcription master regulators induce lineage-specific genes to restrict plasticity, it remains unclear whether plasticity is actively suppressed by lineage-specific repressors. Here we computationally predict so-called safeguard repressors for 18 cell types that block phenotypic plasticity lifelong. We validated hepatocyte-specific candidates using reprogramming, revealing that prospero homeobox protein 1 (PROX1) enhanced hepatocyte identity by direct repression of alternative fate master regulators. In mice, *Prox1* was required for efficient hepatocyte regeneration after injury and was sufficient to prevent liver tumorigenesis. In line with patient data, *Prox1* depletion caused hepatocyte fate loss in vivo and enabled the transition of hepatocellular carcinoma to cholangiocarcinoma. Conversely, overexpression promoted cholangiocarcinoma to hepatocellular carcinoma transdifferentiation. Our findings provide evidence for PROX1 as a hepatocyte-specific safeguard and support a model where cell-type-specific repressors actively suppress plasticity throughout life to safeguard lineage identity and thus prevent disease.

## Abstract (web summary)

This study identifies candidate safeguard repressor transcription factors that repress alternate lineages in mature cell types and provides functional evidence that *Prox1* performs such a function in hepatocytes during reprogramming, regeneration and in cancer.

## Main

Cell fate plasticity is gradually restricted during development, enabling stem cells to generate mature cell types through differentiation^1^. Unlocked cellular plasticity, such as blocked differentiation or dedifferentiation/transdifferentiation, can promote disease and has emerged as a cancer hallmark^2^. While precise mechanisms governing plasticity remain elusive, transcription factors (TFs) are important regulators of this process.

Individual lineage-specific master regulator TFs can activate gene networks to induce specific cell types^3–5^. Their loss in mature cells, for example, *Pax5* in B cells or *Ptf1a* in acinar cells^6,7^, can cause cancer. Transcriptional repressors can also define cell fate^8,9^, as exemplified by RE1 silencing transcription factor (REST), which represses neuronal genes in non-neuronal cells^10^. However, as a principle for maintaining cell fate, one repressor silencing one cell identity would require hundreds of repressors to silence all alternative identities. We recently discovered a cell-type-specific ‘safeguard repressor’ that blocks multiple alternate cell fates. Unlike REST, the neuron-specific TF myelin transcription factor 1 like (MYT1L) binds and represses many non-neuronal genes to promote neuronal identity^11,12^. MYT1L is expressed lifelong, and its loss of function is associated with mental disorders and brain cancer^13,14^. The prevalence of such safeguard repressors across lineages and their role in cancer is unclear.

Here we developed a computational approach to identify safeguard repressors across 18 cell types that promote and maintain cell identity by suppressing cell fate plasticity and cancer. We validated hepatocyte-specific candidates and found prospero homeobox protein 1 (PROX1) to safeguard hepatocyte identity by repressing alternative fate master regulators during reprogramming. In mice, *Prox1* was required for hepatocyte regeneration after injury and sufficient to block liver tumor initiation and progression. Manipulating PROX1 levels can switch transformed hepatocytes between cholangiocarcinoma (CCA) and hepatocellular carcinoma (HCC) fates. This supports a model where cell-type-specific safeguard repressors actively suppress unwanted plasticity to induce and maintain cell identity and block tumorigenesis.

## Results

### Safeguard repressor candidates across 18 cell types

**Fig. 1:**
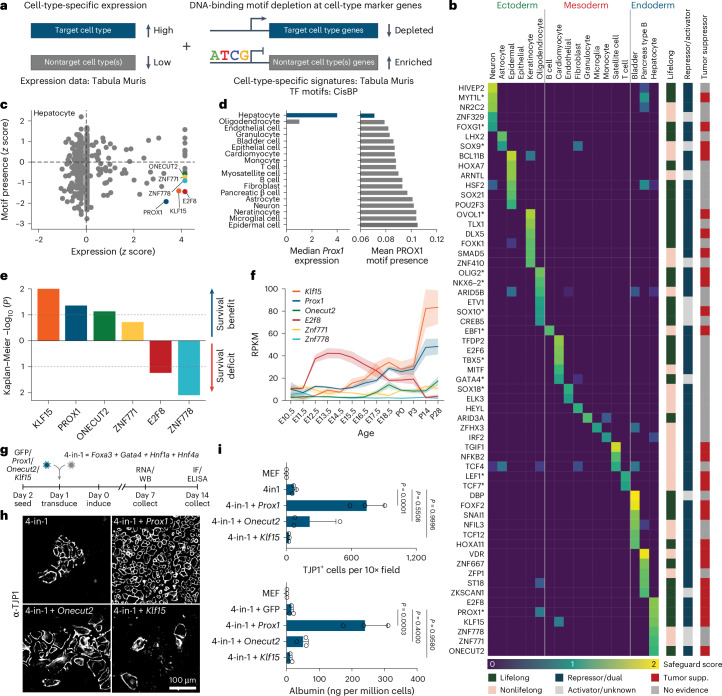
Safeguard repressor screen. **a**, Schematic representation of safeguard repressor prediction based on TF expression and DNA-binding motif analysis. **b**, Scores of top safeguard repressor candidates across 18 cell types, including lifelong expression, repressor/activator activity, tumor suppressor roles and TFs promoting indicated cell fate (asterisks). **c**, TF expression and motif presence analysis highlight top six hepatocyte safeguard repressor candidates. **d**, Prox1 expression (left) and motif counts in signature genes of indicated cell types (right). **e**, log-rank test between Kaplan–Meier curves from patients with HCC in TCGA, segregated by high versus low expression of indicated candidates. **f**, Bulk RNA-seq expression of hepatocyte candidates during mouse liver development^17^. **g**, Validation of top three liver candidates by overexpression using 4-in-1 iHep reprogramming. **h**, TJP1 immunofluorescence of cells in **g** (*n* = 3). **i**, Quantification of TJP1^+^ cells and albumin secretion of cells in **g** (*n* = 3). Scale bar = 100 µm (**h**). Bar and line graphs show mean (*n* = 3), and error bars = s.d. (**f**,**i**). Two-tailed Dunnett’s test (**i**). *P* values are displayed. TCGA, The Cancer Genome Atlas; iHep, induced hepatocyte; RPKM, reads per kilobase million; WB, western blot; IF, immunofluorescence. Source data

**Extended Data Fig. 1:**
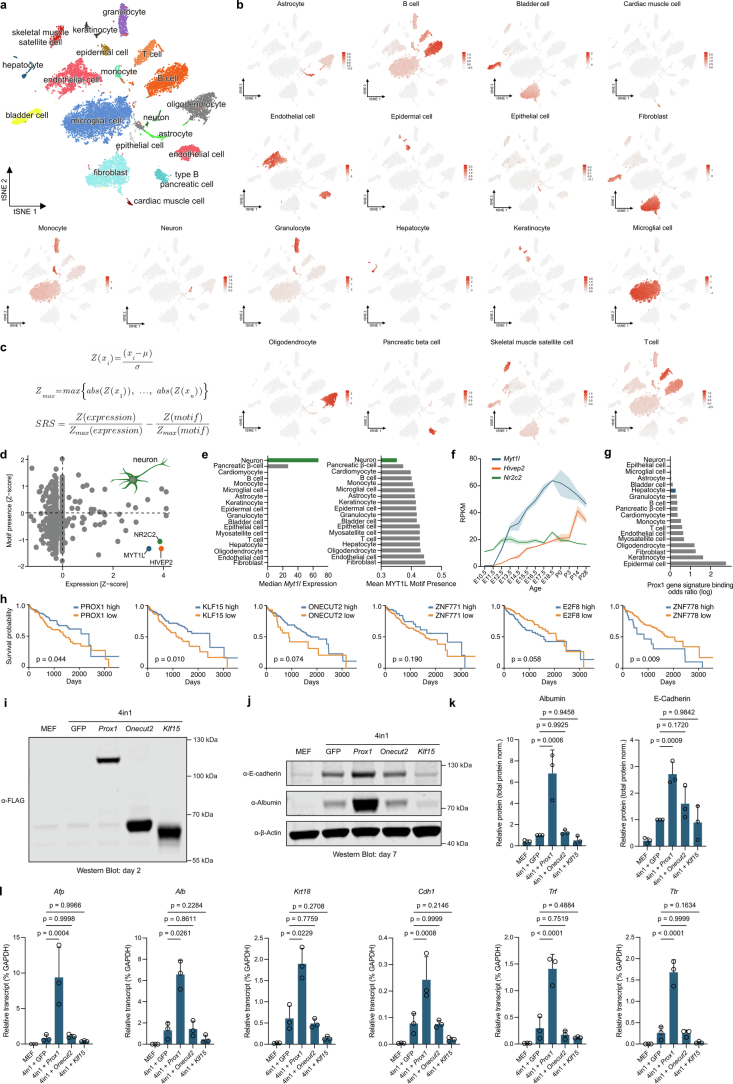
In silico and reprogramming screen identifies safeguard repressors. **a**, Single-cell t-SNE of 18 cell types annotated by the Tabula Muris consortium. **b**, Cell-type-specific gene signatures used in this study displayed across all cells in **a**. **c**, Equations to calculate a safeguard repressor score (SRS) for each TF (see Methods for details). **d**, Safeguard repressor score analysis of 1,296 TFs shows neuronal candidates, including MYT1L. **e**, *Myt1l* expression and number of MYT1L motifs in cell-type-signature promoters. **f**, RNA-seq expression of the top neuronal safeguard repressor candidates in mouse brain development. **g**, Odds ratio of PROX1 CUT&RUN peaks from mouse liver in cell-type-signature gene promoters. **h**, TCGA survival curves for patients with HCC stratified by expression (high/low) of indicated safeguard repressors. **i**, Western blot of FLAG-tagged candidates upon overexpression at day 2 during hepatocyte reprogramming (n = 3). **j**, Western blot of albumin and E-cadherin at day 7 of hepatocyte reprogramming with indicated candidates (n = 3). **k**, Quantification of albumin and E-cadherin protein expression in **j**, normalized to total protein expression (n = 3). **l**, Expression analysis of indicated hepatocyte markers in cells treated as in **j** using qRT-PCR. Bar graphs show mean (n = 3), error bars = SD, two-tailed Dunnett’s test (**k**,**l**) or log-rank test (**h**). p values are displayed. Source data

To identify safeguard repressors, we defined the following three features: (1) cell-type-specific and lifelong expression, (2) binding and repressing alternative fate genes and (3) promoting and maintaining cell identity. We focused on 18 cell types across all germ layers and used Tabula Muris data to define cell-type-specific gene signatures and expression specificity of 1,296 detected TFs^15^. We expected safeguard candidates to bind alternative fate genes and integrated cell-type-specific expression and DNA-binding motif depletion at signature genes to derive a safeguard repressor score (Fig. 1a, Extended Data Fig. 1 and Supplementary Table 1). A searchable database of this analysis is accessible at https://apps.embl.de/safeguard/.

We shortlisted 59 candidates, of which 50 have reported repressor or dual activator/repressor function (Fig. 1b and Supplementary Table 2). In total, 33 exhibited continued expression in 2-year-old mice^16^, and 77% (17/22) of heart, brain and liver candidates were expressed at high levels throughout development^17^. Overall, 27 candidates satisfied our criteria for lifelong safeguard repressors, and 14 were reported to promote the predicted cell fate, including the neuronal safeguard MYT1L^12^ (Fig. 1b and Extended Data Fig. 1d–f). The top hepatocyte candidate, PROX1, exhibited hepatocyte-specific expression and bound many nonhepatocyte genes, based on motif enrichment and cleavage under targets and release using nuclease (CUT&RUN) binding (Fig. 1c,d, Extended Data Fig. 1g and Supplementary Table 3). Interestingly, 59% (16/27) of the candidates are reported to exhibit tumor-suppressive roles in their respective cell types (Fig. 1b).

### Patient data indicate PROX1 as liver safeguard

**Fig. 2:**
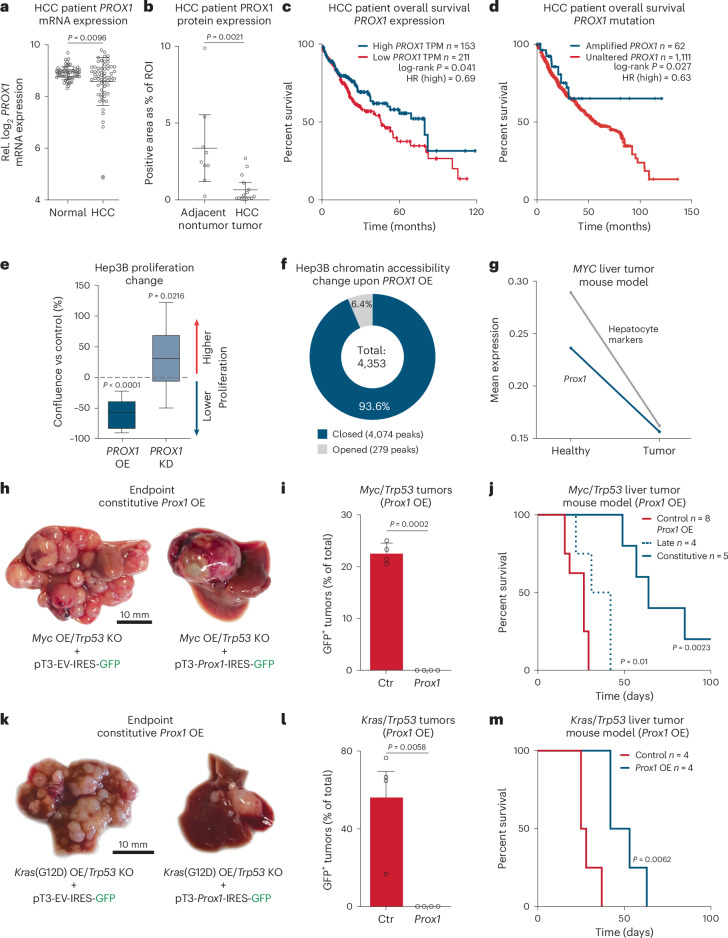
PROX1 suppresses liver cancer formation and progression. **a**, *PROX1* expression in tumors from patients with HCC and paired normal tissue^24^. **b**, PROX1 protein in patients with HCC liver tumors and adjacent nontumor tissues (*n* = 15). **c**, Survival of patients with HCC stratified by *PROX1* expression levels (40% high-expression cutoff)^32^. **d**, Survival of patients with HCC ranked by *PROX1* amplification status^25–31^. **e**, Confluency of Hep3B cancer cells upon *PROX1* shRNA-KD or OE for 7 days normalized to uninduced controls (*n* = 3). **f**, Differential ATAC–seq accessibility in Hep3B cells upon *PROX1* OE for 2 days (*n* = 3; adjusted *P* < 0.05). **g**, *Prox1* and hepatocyte signature expression across 7,793 single cells in healthy (day 0) and *MYC*-induced mouse HCC model (day 28)^35^. **h**, Mouse livers following HDTVI-mediated *Myc* OE and *Trp53* KO with constitutive *Prox1* OE (*n* = 5). **i**, Percentage of GFP^+^ tumors in mice treated as in **h** (*n* = 4). **j**, Survival of mice treated as in **h** following constitutive *Prox1* OE (*n* = 5) or doxycycline-inducible late *Prox1* OE (*n* = 4) at day 14 compared to control (*n* = 5 for constitutive OE and *n* = 3 for late OE). **k**, HDTVI-induced liver tumors following *Kras*(G12D) OE and *Trp53* KO with constitutive *Prox1* OE (OE; *n* = 4). **l**, Percentage of GFP^+^ tumors in mice treated as in **k** (*n* = 4). **m**, Survival of mice treated as in **k** following constitutive *Prox1* OE or control (*n* = 4). Scale bar = 10 mm (**h**,**k**). Bar graphs and scatter plots show mean, error bars = s.d., boxplots show median and IQR and whiskers = 1.5× IQR from specified replicates. Unpaired two-tailed *t* test (**a**,**b**,**i**,**l**), log-rank test (**c**,**d**,**j**,**m**) and two-tailed one-sample *t* test (**e**). *P* values are displayed. ROI, region of interest; HR, hazard ratio; KO, knockout; KD, knockdown; OE, overexpression; IQR, interquartile range. Source data

**Extended Data Fig. 2:**
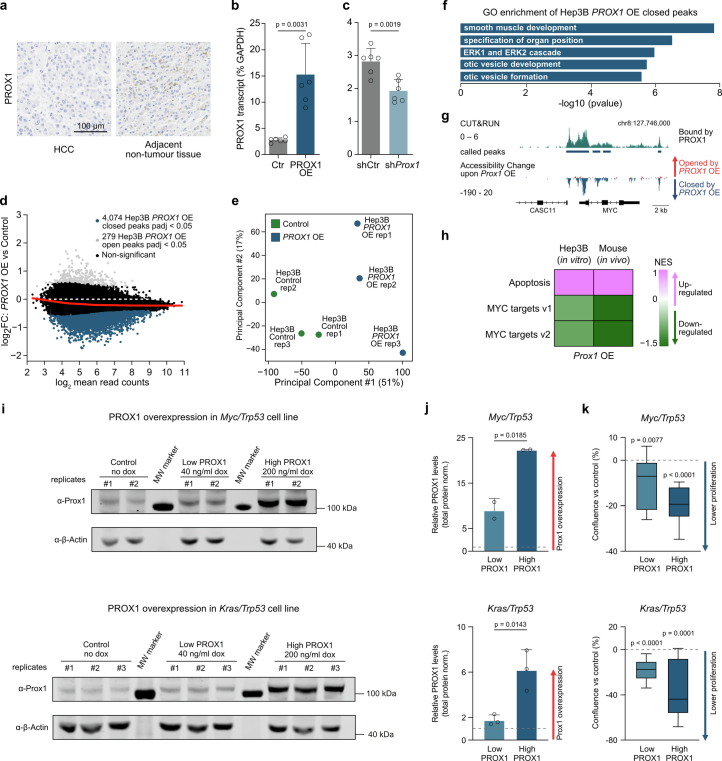
PROX1 induces chromatin closure and growth delays in mouse HCC cell lines. **a**, PROX1 protein level in liver sections from patients with HCC (n ≥ 3). **b**,**c**, Inducible *PROX1* overexpression (OE; **b**) and knockdown (shPROX1; **c**) in Hep3B cells at day 2 compared to control (Ctr) by qRT-PCR (n = 6 each). **d**, MA-plot of ATAC-seq differentially accessible regions (DARs) upon *PROX1* OE in Hep3B cells at day 2 (n = 3). **e**, PCA of DARs in **d** labeled by condition and replicate. **f**, Genomic annotations of chromatin peaks closed upon *PROX1* OE compared to control determined by GREAT. **g**, IGV tracks of PROX1 binding by CUT&RUN and ATAC-seq accessibility change at the *MYC* locus in Hep3B cells 2 days after *PROX1* OE compared to control (n = 3 each). **h**, GSEA normalized enrichment scores (NES) for MYC targets and apoptosis upon inducible *PROX1* OE for 7 days in Hep3B cells or 2 days in *Myc*/*Trp53* HCC mouse models (day 16 collection; n = 3 each). **i**, Doxycycline dose-dependent *Prox1* OE in mouse tumor-derived cell lines transformed with *Trp53* KO with OE of *Myc* (*Myc*/*Trp53*; n = 2) or *Kras*(G12D) (*Kras*/*Trp53*; n = 3) determined by western blot at day 3. **j**, Quantification of PROX1 protein levels of cells treated as in **i**. **k**, Confluency of cells treated as in **i** normalized to uninduced controls. Scale bar = 100 µm (**a**). Bar graphs show mean with error bars = SD, boxplots show median and interquartile range (IQR), whiskers = 1.5× IQR, from indicated biological replicates. Two-sided t test (**b**,**c**), unpaired t test (**j**) or one-sample t test (**k**). P values are displayed. Source data

Cell fate loss has a crucial role in liver disease, including liver cancer^18,19^. High expression of the hepatocyte candidates (*PROX1*, *KLF15, ONECUT2* and *ZNF771*) correlated with better prognosis in patients with HCC (Fig. 1e and Extended Data Fig. 1h). However, only *Prox1*, *Onecut2* and *Klf15* showed lifelong expression in the liver (Fig. 1b,f). To test whether they promote hepatocyte identity, we overexpressed them in mouse embryonic fibroblasts (MEFs) during reprogramming toward hepatocytes^20^ (Fig. 1g). PROX1 increased hepatocyte-like cell induction greater than tenfold, as measured by TJP1 protein expression, elevated albumin secretion per cell 17-fold, and boosted expression of hepatocyte-specific markers (Fig. 1h,i and Extended Data Fig. 1i–l). In mice, PROX1 promotes liver development^21^, but both tumor-promoting and suppressor roles have been reported in liver cancer models^22,23^. Hence, we assessed whether *PROX1* is dysregulated in patients with HCC and found lower expression in tumor samples than in normal tissues^24^ (Fig. 2a). Immunohistology confirmed reduced PROX1 levels in HCC compared to adjacent tissue (Fig. 2b and Extended Data Fig. 2a). Furthermore, in patients with HCC, high *PROX1* expression or chromosomal amplifications including *PROX1* were both associated with increased survival^25–32^ (Fig. 2c,d). These findings suggest that PROX1 has a tumor-suppressive role in HCC.

To investigate the effect of PROX1 in human HCC cells, we generated Hep3B cell lines with inducible *PROX1* overexpression (OE) or knockdown (KO) (Extended Data Fig. 2b,c). While *PROX1* OE decreased proliferation by 60%, shRNA-mediated depletion enhanced proliferation in vitro (Fig. 2e). We characterized chromatin organization upon *Prox1* OE using assay for transposase-accessible chromatin with sequencing (ATAC–seq) and found 4,353 differentially accessible peaks (adjusted *P* < 0.05), of which 4,074 (93.6%) were closed compared to control (Fig. 2f, Extended Data Fig. 2d,e and Supplementary Table 4). Several nonhepatocyte terms, such as smooth muscle development and pro-proliferative signaling pathways, were enriched in regions closed by *Prox1* (Extended Data Fig. 2f). To identify PROX1 target genes, we conducted CUT&RUN upon *PROX1* OE (Supplementary Fig. 1), revealing 16,183 peaks harboring a PROX1 motif, and defined direct PROX1 target genes within 2 kb of a transcription start site (TSS). Most peaks closed upon OE (Fisher test, *P* < 2.2 × 10^−16^, odds ratio 1.6), including the MYC locus. RNA-seq and gene regulatory network analysis confirmed that *MYC* and MYC targets were downregulated upon *PROX1* OE, coinciding with increased apoptosis signatures (Extended Data Fig. 2g,h and Supplementary Table 5), suggesting PROX1 could directly suppress pro-proliferative pathways. To investigate dose-dependent effects, we introduced doxycycline-inducible *Prox1* in two cell lines derived from mouse tumors driven by *Trp53* KO with either *Myc* or *Kras*(G12D) OE^33^. In both, we observed decreased proliferation in a PROX1 dose-dependent manner (Extended Data Fig. 2i–k). This shows that PROX1 primarily closes chromatin in liver cancer cells and reduces their proliferation by gene repression.

### PROX1 blocks liver cancer in mice

**Extended Data Fig. 3:**
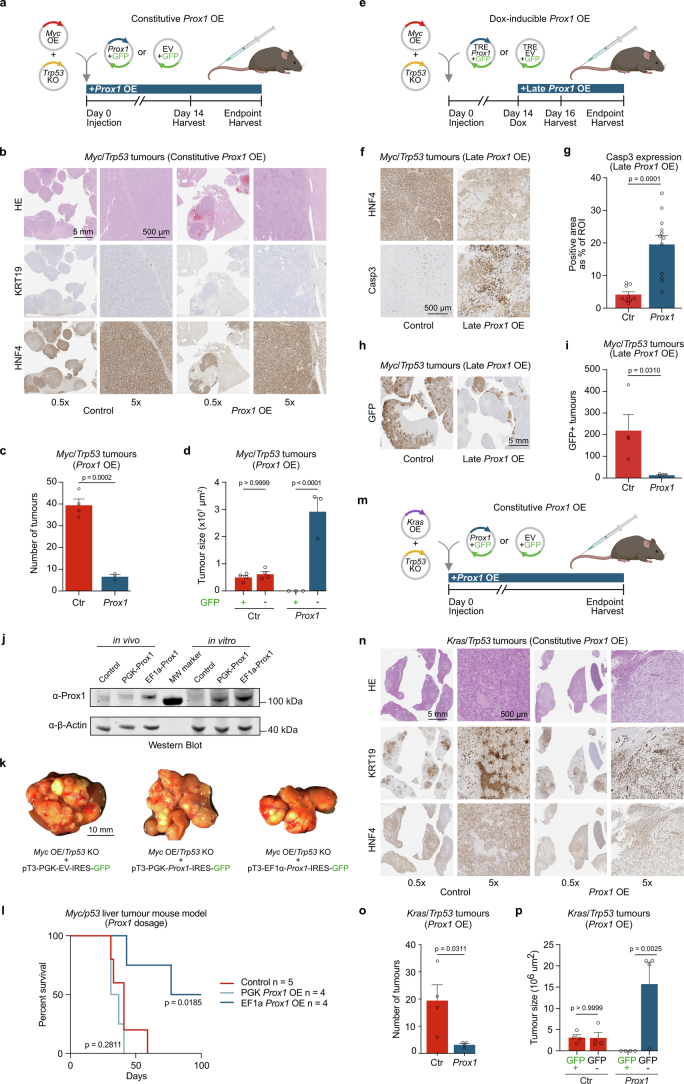
PROX1 prevents liver cancer induction and progression in mice. **a**, HCC-like tumor induction by *Myc* OE and *Trp53* KO using HDTVI with constitutive *Prox1* OE or GFP controls. **b**, Hematoxylin and eosin (H&E), HNF4 and KRT19 histology of livers treated as in **a** (n ≥ 3). **c**, Tumor numbers upon treatment as in **a**, following OE of *Prox1*-IRES-GFP (n = 3) vs GFP control (n = 4). **d**, Size of tumors (cross-sectional area) in **c** with or without GFP expression indicating transgene expression. **e**, Schematic of doxycycline-inducible late *Prox1* OE at day 14 following HDTVI-tumor induction as in **a**. **f**, Livers treated as in **e** following histological staining for HNF4 and CASP3 at day 16 (n ≥ 3). **g**, Quantification of CASP3 levels in GFP^+^ tumors in **f** following late *Prox1* OE (n = 3) vs GFP control (n = 4). **h**, GFP staining in livers treated as in **e** at the endpoint. **i**, Quantification of GFP^+^ tumor nodule numbers shown in **h** (n = 4). **j**, Western blot of PROX1 following PGK- and EF1a-promoter driven OE in vivo and in vitro compared to controls. **k**, Mouse livers following HDTVI to induce *Myc* OE and *Trp53* KO with *Prox1* OE using PGK- or EF1a-promoters (n = 4) compared to PGK-GFP controls (n = 5). **l**, Overall survival of mice treated as in **k**. **m**, Tumor induction via HDTVI-mediated *Kras*(G12D) OE and *Trp53* KO with constitutive *Prox1* OE or GFP controls. **n**, Liver histology following treatment as in **m** stained with H&E, HNF4 and KRT19 (n ≥ 3). **o**, Tumor numbers upon treatment as in **m** following OE of *Prox1*-IRES-GFP (n = 4) vs GFP control (n = 4). **p**, Size of tumors (cross-sectional area) in **o** with or without GFP expression indicating transgene expression. Scale bars = 10 mm, 5 mm and 500 µm (**k**,**b**,**f**,**h**,**n**). Bar graphs show mean values from indicated biological replicates, error bars = SD. Unpaired two-tailed t test (**c**,**d**,**g**,**i**,**o**,**p**), Bonferroni correction (**d**,**p**) and log-rank test (**l**). p values are displayed. The figure is created with BioRender.com. Source data

Next, we analyzed *Prox1* expression in single-cell RNA-seq data of an HCC mouse model^34^. We found lower *Prox1* levels in transformed cells compared to hepatocytes, coinciding with decreased hepatocyte identity (Fig. 2g). To investigate whether manipulating PROX1 can prevent liver tumor formation, we used a HCC mouse model combining *Myc* OE and *Trp53* KO (*Myc*/*Trp53*) via hydrodynamic tail-vein injection (HDTVI; Fig. 2h, Extended Data Fig. 3a,b and Supplementary Table 6). After 2 weeks, mice developed carcinomas resembling HCC with the expression of hepatocyte nuclear factor-4α (HNF4α) that lacked glandular structures and keratin 19 (KRT19) expression typical for CCAs. Constitutive overexpression of *Prox1*-IRES-GFP led to fewer tumor nodules at the endpoint (6.7 versus 39.5 nodules), and all resulting tumors were GFP-negative, indicating selection against *Prox1* OE (Fig. 2i and Extended Data Fig. 3c,d). Indeed, *Prox1* overexpression increased median survival from 29 to 64 days, with several mice surviving the 100-day experiment (Fig. 2j and Extended Data Fig. 3l). We found that only high PROX1 levels (driven by the EF1a promoter) significantly increased survival (Extended Data Fig. 3j–l).

**Extended Data Fig. 4:**
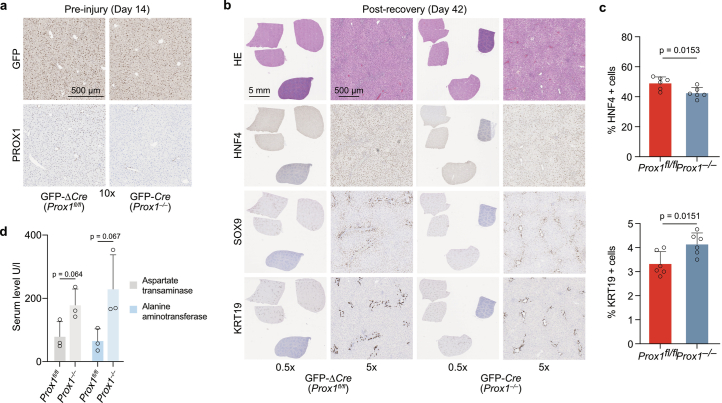
Impaired regeneration upon DDC-liver injury in Prox1-deleted mice. **a**, Mouse liver histology stained for PROX1 and GFP 2 weeks following AAV GFP-*Cre*-mediated *Prox1* deletion in conditional *Prox1* knockout mice (*Prox1*^*fl*/*fl*^) compared GFP-Δ*Cre* control mice before injury induction (n = 3). **b**, H&E, HNF4, SOX9 and KRT19 liver staining treated as in **a** and following 2 weeks of DDC diet and 2 weeks of recovery with normal diet (n = 3). **c**, Percentage of HNF4^+^ and KRT19^+^ cells in liver sections from mice treated as in **b** (n = 3). **d**, Serum levels of aspartate transaminase and alanine aminotransferase from mice treated as in **b** (n = 3). Scale bar = 5 mm and 500 µm (**a**,**b**). Bar graphs show mean (n = 3), error bars = SD and unpaired two-sided t test (**c,d**). P values are displayed. Source data

We combined the same model with doxycycline-inducible *Prox1* OE to test the effect after tumor nodules had formed over 14 days post-HDTVI (Extended Data Fig. 3e). Two days following doxycycline treatment, we observed a greater than fourfold increase in apoptosis in tumors as judged by CASP3 histology (Extended Data Fig. 3f,g). RNA-seq confirmed the upregulation of apoptosis and downregulation of pro-proliferative MYC signatures, mirroring the findings in Hep3B cells (Extended Data Fig. 2h and Supplementary Table 7). Notably, late *Prox1* OE reduced GFP^+^ tumor nodules at the endpoint (219.5 versus 13.75 nodules) and significantly increased median survival from 17 to 36.5 days (Fig. 2j and Extended Data Fig. 3h,i), suggesting that PROX1 blocks tumor progression. To determine whether PROX1 suppresses cancers of different genetic aetiologies, we used a second mouse model induced by HDTVI-mediated *Kras*(G12D) OE and *Trp53* KO (*Kras*/*Trp53*; Extended Data Fig. 3m). This model presented both HCC and CCA features, with morphologically complex tumors, lower HNF4α and higher KRT19 staining (Extended Data Fig. 3n). Here *Prox1* OE also reduced the number of tumor nodules (3.25 versus 19.5 nodules), all lacking Prox1-IRES-GFP, and extended survival from 26.5 to 47.5 days (Fig. 2k–m and Extended Data Fig. 4o,p). This demonstrates that PROX1 functions as a tumor suppressor by impeding tumor initiation and progression in distinct liver cancer mouse models.

### PROX1 is required for liver regeneration and reprogramming

**Fig. 3:**
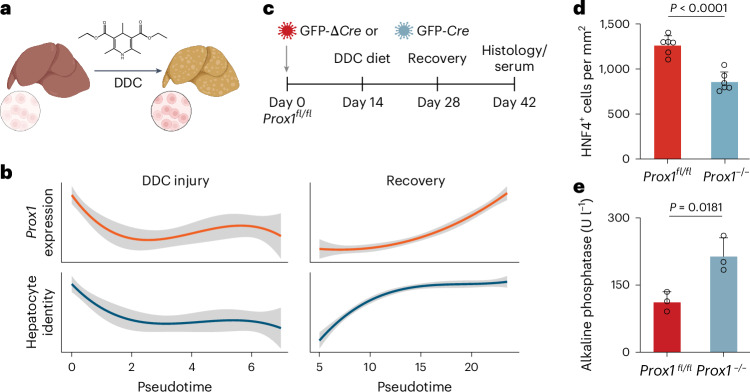
PROX1 is necessary during liver regeneration. **a**, Schematic representation of DDC diet-induced liver injury and regeneration in mice. The figure is created with BioRender.com. **b**, Pseudotime-ordered RNA expression of *Prox1* and hepatocyte signature of mice treated as in **a**, across 1,866 single cells^35^. **c**, Liver injury and recovery as in **a** following *Cre*-mediated *Prox1* deletion in conditional *Prox1*^*fl*/*fl*^ knockout mice. **d**, Number of HNF4^+^ cells per area in livers from mice treated as in **c** (*n* = 3). **e**, Serum levels of ALP from mice treated as in **c** (*n* = 3). Bar graphs show mean (*n* = 3), error bars = s.d., and unpaired two-tailed *t* test. *P* values are displayed. Source data

Cell fate plasticity plays a key role in other fate transitions, such as in regeneration following injury or direct cell reprogramming. Upon liver injury, mature hepatocytes can dedifferentiate by reactivating progenitor-like programs, followed by proliferation and regeneration of hepatocytes. Using single-cell RNA-seq data^35^, we found that *Prox1* expression and hepatocyte identity sharply decreased following 3,5-diethoxycarbonyl-1,4-dihydrocollidine (DDC)-induced injury and recovered during regeneration (Fig. 3a,b). We therefore treated conditional *Prox1* knockout mice^36^ with DDC and found that *Cre*-mediated homozygous *Prox1*^*−*^/^*−*^ deletion led to a reduction in the number and density of HNF4^+^ hepatocytes by ~33%, while the percentage of KRT19^+^ cholangiocytes increased following regeneration (Fig. 3c,d and Extended Data Fig. 4a–c). Following recovery, serum levels of the liver injury marker alkaline phosphatase (ALP) were elevated twofold in *Prox1*-deleted mice, while other markers increased without reaching statistical significance (Fig. 3e and Extended Data Fig. 4d), suggesting that PROX1 is required for efficient hepatocyte regeneration after injury.

**Fig. 4:**
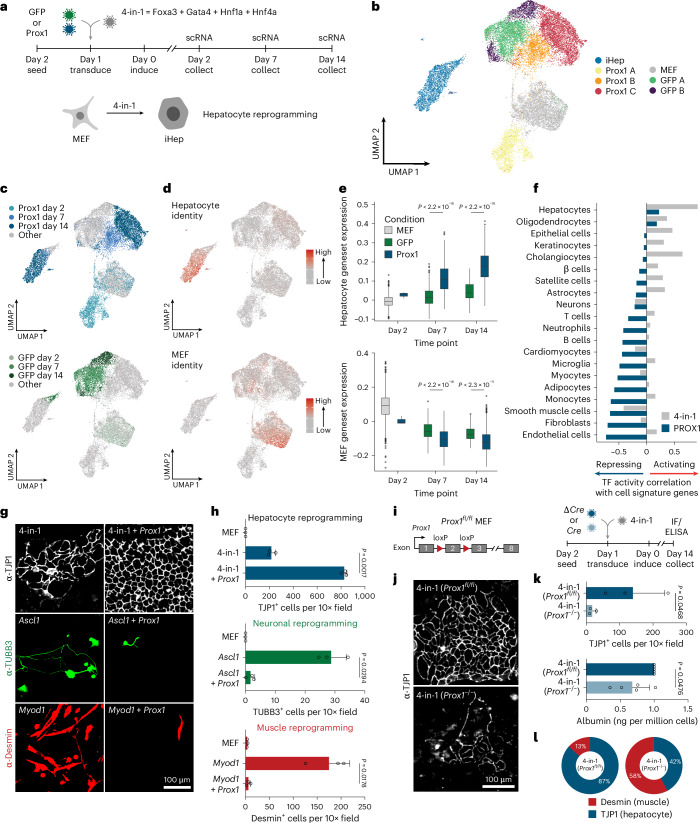
PROX1 promotes hepatocyte cell fate via multilineage repression. **a**, Hepatocyte reprogramming time course with or without *Prox1* OE analyzed by single-cell RNA-seq. **b**, A total of 22,761 cells treated as in **a**, following clustering and UMAP projection (*n* = 2). **c**, Annotation of cells in **b** based on experimental treatment and time point. **d**, Projection of hepatocyte and fibroblast identity onto cells in **b**. **e**, Hepatocyte and fibroblast identity scores in iHep cluster from **b**. **f**, Correlation of various cell identity scores with 4-in-1 or PROX1 regulon activity. **g**, Immunofluorescence of reprogrammed hepatocytes (4-in-1), neurons (*Ascl1*) or myocytes (*Myod1*) with or without *Prox1* OE stained for TJP1 (hepatocyte), TUBB3 (neuronal) or desmin (myocyte) at day 14 (*n* = 3). **h**, Immunofluorescence quantification of cells in **g** (*n* = 3). **i**, *Prox1* KO during iHep reprogramming via *Cre*-mediated deletion in *Prox1*^*fl*/*fl*^ MEFs. **j**, TJP1 immunofluorescence of cells in **i**) at day 14 (*n* = 3). **k**, Number of TJP1^+^ cells (*n* = 3) and albumin secretion (*n* = 5) of cells in **i**. **l**, Proportion of reprogrammed cells in **i**, positive for desmin or TJP1 (*n* = 3). Scale bar = 100 µm (**g**,**j**). Bar graphs show mean, error bars = s.d., boxplots show median and IQR, whiskers = 1.5× IQR, from specified replicates. and unpaired two-tailed *t* test. *P* values are displayed. Source data

To investigate how PROX1 could enhance hepatocyte fate, we assessed the effects of *Prox1* during 4-in-1-induced hepatocyte reprogramming of MEFs using scRNA-seq on days 2, 7 and 14 (Fig. 4a and Supplementary Fig. 2a–d). *Prox1*-overexpressing cells largely constituted distinct clusters corresponding with experimental time points (Fig. 4b,c). We scored each cell for cell-type-specific gene signatures and found one MEF- and one hepatocyte-like cluster (Fig. 4d). *Prox1* OE increased hepatocyte identity, and the number of successfully reprogrammed hepatocytes was greater than sevenfold. These cells also downregulated the MEF fate more efficiently and displayed fewer alternative cell identities (Fig. 4e and Supplementary Fig. 2e–i). Correspondingly, the activity of a PROX1 regulon, defined based on PROX1 CUT&RUN data in MEFs, negatively correlated with the fibroblast identity signature, suggesting that PROX1 directly represses MEF genes (Fig. 4f, Supplementary Fig. 1e and Supplementary Table 8). Interestingly, while the regulon activity of the liver inducers (4-in-1) correlated positively with hepatocyte and alternative cell identities, such as cholangiocytes, PROX1 activity negatively correlated with all tested cell identities except for hepatocyte and oligodendrocyte signatures (Fig. 4f). This suggests that PROX1 can directly repress nonhepatic gene signatures, potentially activated by 4-in-1, to promote the desired hepatocyte fate.

### PROX1 blocks alternative fates during hepatocyte reprogramming

We next tested the effect of PROX1 OE on neuronal^37^ and myocyte reprogramming^38^. Both were almost entirely abolished upon *Prox1* OE, as determined by neuronal (TUBB3) and myocyte (desmin) marker protein expression (Fig. 4g,h and Supplementary Fig. 3 (western blots and gels for Supplementary Figs. 3a–c and 4a are provided in the Source Data section). Interestingly, while MYOD1 alone did not induce any liver-like cells, 18% of MYOD1-reprogrammed cells expressed TJP1 upon *Prox1* co-expression (Supplementary Fig. 3f). MYT1L had similar effects, inhibiting myocyte and hepatocyte reprogramming while promoting neuronal conversion (Supplementary Fig. 3). This suggests that safeguard repressors such as PROX1 and MYT1L promote a specific cell fate and might redirect the cell fates induced by neuronal and muscle master regulators.

**Extended Data Fig. 5:**
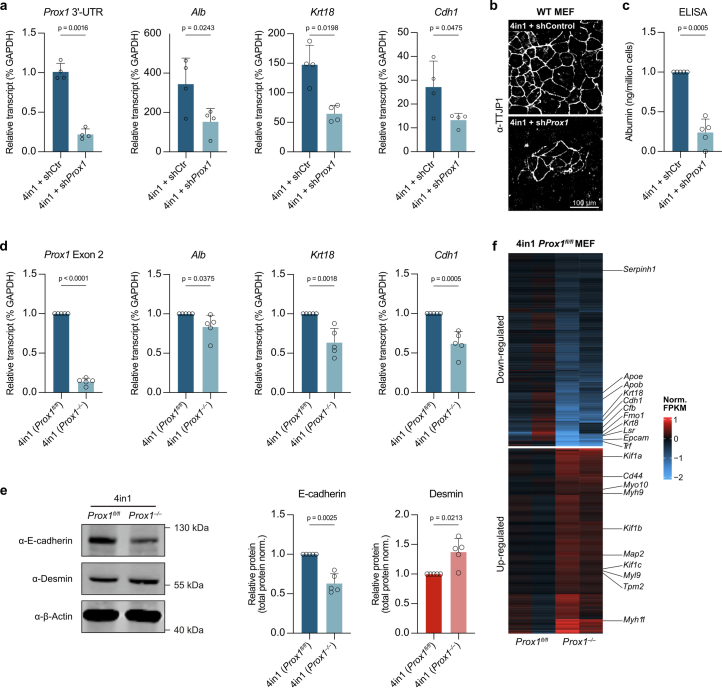
Depletion of Prox1 reduces hepatocyte reprogramming efficiency and fidelity. **a**, Expression of *Prox1* and hepatocyte markers at day 14 of iHep reprogramming upon *Prox1* or control shRNA treatment based on qRT-PCR. **b**,**c**, TJP1 immunofluorescence (**b**) and normalized albumin secretion (**c**) of cells in **a**. **d**, Expression of indicated genes at day 14 of iHep reprogramming using *Prox1*^*fl*/*fl*^ MEFs upon treatment with *Cre* (*Prox1*^−/−^) or Δ*Cre* (*Prox1*^*fl*/*fl*^) based on qRT-PCR. **e**, E-cadherin (hepatocyte) and desmin (muscle) protein quantification of cells in **d** based on western blot analysis. **f**, Differential gene expression of cells in **d** based on RNA-seq (n = 2). Scale bar = 100 µm (**b**). Bar graphs show mean of n = 4 (**a**,**b**) or n = 5 (**c**–**e**) biological replicates, error bars = SD and two-tailed t test. p values are displayed. Source data

Because *Prox1* OE was sufficient to suppress alternative fates, we determined whether endogenous *Prox1* expression is necessary for hepatocyte reprogramming. Upon shRNA-mediated *Prox1* knockdown, albumin secretion, TJP1 immunofluorescence and hepatocyte gene expression indicated impaired liver reprogramming (Extended Data Fig. 5). Similarly, conditional *Prox1* deletion resulted in greater than sevenfold decreased TJP1-positive hepatocyte-like cells and lower albumin secretion per cell (Fig. 4i–k and Extended Data Fig. 5d). Strikingly, the fraction of desmin-expressing cells during liver reprogramming increased upon *Prox1* deletion (Fig. 4l and Extended Data Fig. 5e). RNA-seq confirmed that genetic *Prox1*^−^^/*−*^ deletion decreased liver marker expression during hepatocyte reprogramming and increased levels of alternative fate markers such as neuronal *Map2* and myocyte *Myh9* (Extended Data Fig. 5f), indicating that PROX1 is sufficient and necessary for efficient hepatocyte fate induction by repressing alternative fates.

### Repression of PROX1 target genes enhances liver fate

**Fig. 5:**
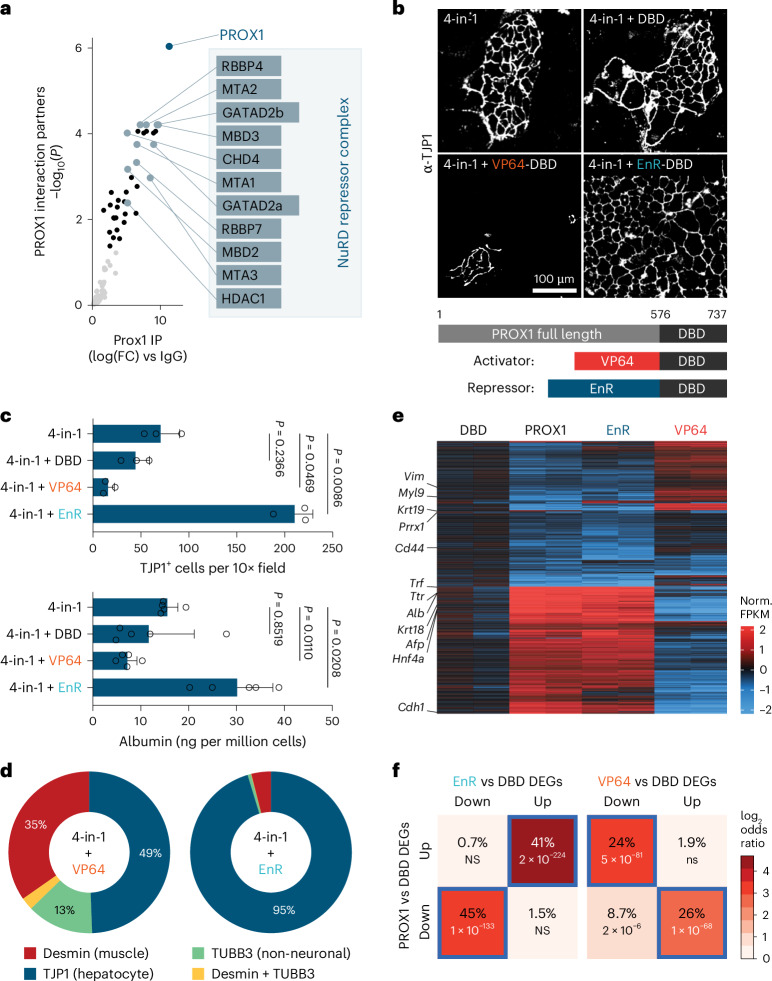
Direct repression of alternative fates by PROX1. **a**, PROX1 interaction partners by mass spectrometry upon immunoprecipitation from mouse liver (*n* = 4). **b**, TJP1 immunofluorescence upon 4-in-1 iHep reprogramming with indicated PROX1 DBD fusion constructs at day 14 (*n* = 3). **c**, Number of TJP1^+^ cells (*n* = 3) and albumin secretion (*n* = 5) of cells in **b**. **d**, Proportion of reprogrammed cells in **b**, positive for indicated cell-type markers and morphology (*n* = 3). **e**, RNA-seq of differentially expressed genes from cells in **b** compared to DBD as a control at day 7 (*n* = 2). **f**, Percent overlap of upregulated and downregulated genes in **e** (*n* = 2). Scale bar = 100 µm (**b**). Bar graphs show mean from specified replicates, and error bars = s.d. Two-tailed Dunnett’s test (**c**), two-tailed Fisher’s exact test (**f**) and *P* values are displayed. FPKM, fragments per kilobase million; NS, not significant. Source data

**Extended Data Fig. 6:**
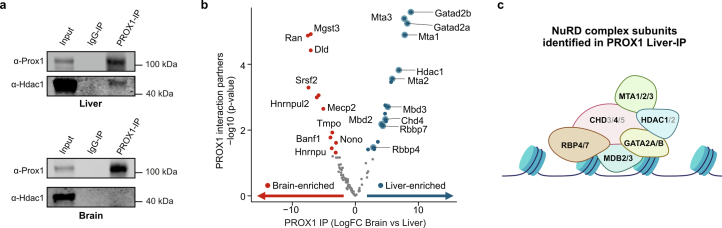
PROX1 interacts with the repressive NuRD complex in liver but not in hippocampus. **a**, Immunoprecipitation (IP) of PROX1 from primary mouse liver and hippocampus compared to IgG control followed by western blot using indicated antibodies (n = 4 each). **b**, Mass spectrometric identification and analysis of differential PROX1 interaction partners between hippocampus and liver (n = 4 each; Methods). **c**, Cartoon of the repressive NuRD complex highlighting liver-specific PROX1 interaction partners in black. The figure is created with BioRender.com. Source data

In mice, *Prox1* is expressed in some neural stem cells to promote neurogenesis^39,40^ and is a key regulator of lymphatic endothelial cell fate^41,42^. There, PROX1 mainly activates gene expression in combination with coactivators such as NR2F2 (refs. ^43,44^). In liver, PROX1 interacts with corepressors, such as histone deacetylases^45^. We performed immunoprecipitation followed by mass spectrometry to identify PROX1 interaction partners in primary mouse liver and brain tissue. In liver, but not in brain, PROX1 interacted with 11 of 14 members of the repressive nucleosome remodeling and deacetylase (NuRD) complex (Fig. 5a, Extended Data Fig. 6 and Supplementary Table 9).

To uncouple cofactor-dependent effects, we fused the DNA-binding domain (DBD) of PROX1 to the VP64 transcriptional activator or the Engrailed repressor (EnR) to directly activate or repress PROX1 target genes (Fig. 5b). The DBD alone did not affect hepatocyte reprogramming. The repressor fusion improved hepatocyte fate induction similarly to full-length PROX1, while the activator fusion had a dominant negative effect, impairing conversion (Fig. 5c and Supplementary Fig. 4a–c). The repressor fusion decreased the fraction of reprogrammed cells that expressed neuronal or myocyte markers, while the activator fusion increased them (Fig. 5d and Supplementary Fig. 4d). We also tested the effects of fusions in neuronal and myocyte reprogramming. As expected, the repressor fusion reduced myocyte and neuronal induction. Interestingly, the activator fusion reduced neuronal cell induction but enhanced myocyte induction, increasing the fraction of desmin-positive cells in both conversions relative to DBD (Supplementary Fig. 4). In hepatocyte reprogramming, the EnR fusion triggered a similar gene expression response as full-length PROX1, inducing hepatocyte genes like *Alb* and repressing nonhepatocyte markers such as *Krt19*, while the activator fusion had the opposite effect (Fig. 5e,f and Supplementary Fig. 4e). This shows that PROX1 represses nonhepatocyte fate genes to promote hepatocyte identity.

### PROX1 closes chromatin at alternative fate signature genes

**Fig. 6:**
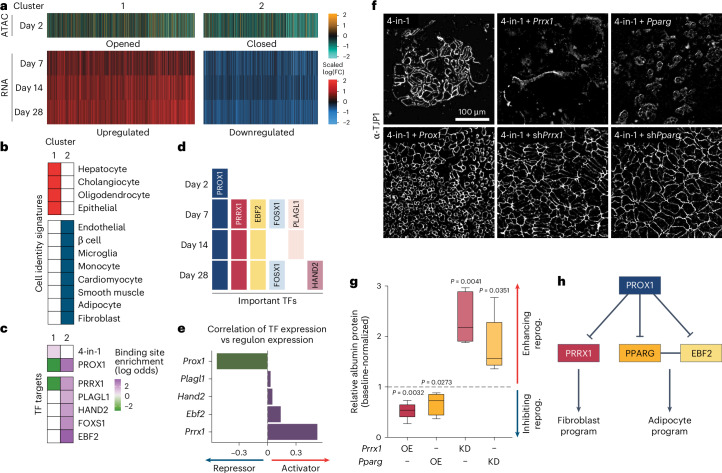
Alternative master regulators are silenced by PROX1. **a**, Hepatocyte reprogramming time course with or without *Prox1* OE clustered based on RNA-seq expression (*n* = 2) and ATAC–seq accessibility (*n* = 3) displayed as scaled log(FC) compared to control. **b**, Overlap of cell signature markers with genes in clusters from **a** (adjusted *P* < 0.01). **c**, Enrichment or depletion of TF targets in promoters of genes clustered in **a** (adjusted *P* < 0.05). **d**, Prediction of key TFs downstream of PROX1 in **a**. **e**, Pearson correlation of indicated TF levels and expression of their target genes in **a** predict activator versus repressor activity. **f**, TJP1 immunofluorescence of 4-in-1 iHep reprogramming with OE or shRNA-mediated KD of *Prrx1* or *Pparg* at day 14 (*n* = 3). **g**, Albumin western blot quantification of cells in **f** normalized to controls (*n* = 5). **h**, Proposed PROX1 gene regulatory network in iHep reprogramming. Scale bar = 100 µm (**f**). Bar graphs show mean, boxplots show median and IQR and whiskers = 1.5× IQR from specified replicates. Benjamini–Hochberg-adjusted two-tailed Fisher’s exact test (**b**,**c**), unpaired two-tailed *t* test (**g**) and *P* values are displayed. Source data

**Extended Data Fig. 7:**
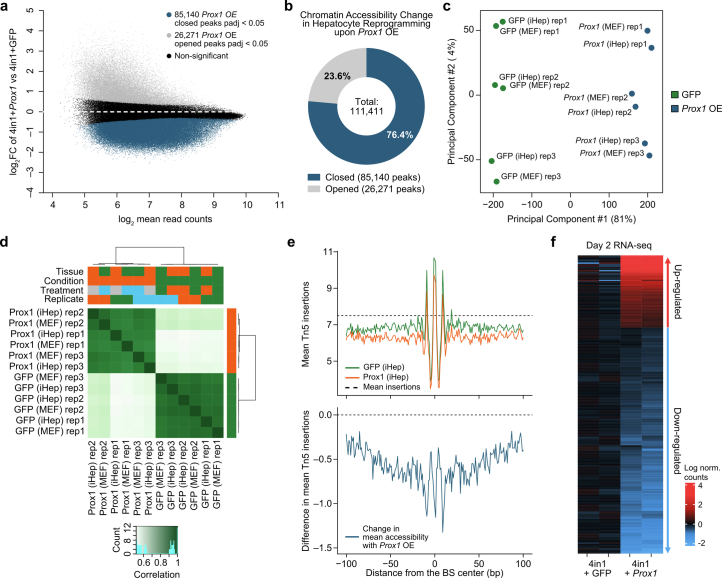
PROX1 predominantly closes bound chromatin and silences associated genes. **a**, MA-plot of ATAC-seq differentially accessible regions (DAR) at day 2 of iHep reprogramming with or without *Prox1* OE (n = 3; Methods). **b**, Percentage of closed and opened regions upon *Prox1* OE in **a**. **c**, PCA of DARs in **a** labeled by condition and replicate and upon OE of *GFP* or *Prox1* alone in MEFs (n = 3). **d**, Correlation of DARs between indicated conditions and replicates in **c**. **e**, Top: mean Tn5 transposon adapter insertions at indicated conditions from **a** centered at PROX1 CUT&RUN binding sites. Bottom: chromatin accessibility changes at PROX1 binding sites between 4-in-1 + GFP and 4-in-1 + *Prox1* indicate decreased accessibility upon *Prox1* OE. **f**, Differential gene expression of cells in **a** based on RNA-seq (n = 2), for genes with differentially accessible promoter (p adj < 0.05) within ±2 kb of their TSS and an overlapping CUT&RUN peak with a PROX1 binding motif (Methods). Source data

To investigate the effects of PROX1 on chromatin organization, we performed ATAC–seq during hepatocyte reprogramming. We found 111,411 differentially accessible peaks (adjusted *P* < 0.05) upon *Prox1* OE, of which 85,140 (76.4%) were closed (Fig. 6a and Extended Data Fig. 7a,b). *Prox1* OE without 4-in-1 caused the closure of 77% of chromatin regions (Extended Data Fig. 7c,d). Using our PROX1 CUT&RUN data in MEFs, we found decreased accessibility at PROX1-bound sites (Extended Data Fig. 7e). Furthermore, 74% of genes with PROX1-bound and differentially accessible promoters were downregulated upon *Prox1* OE (Extended Data Fig. 7f), showing that PROX1 primarily closes chromatin and reduces the expression of directly bound target gene promoters.

To characterize PROX1 target genes during reprogramming, we performed bulk RNA-seq on days 7, 14 and 28. Based on promoter accessibility and differential expression following *Prox1* OE, these grouped into two clusters (Fig. 6a). Cluster 1 (hepatocyte cluster) contained 3,629 upregulated genes with increased promoter accessibility and was enriched for hepatocyte identity genes but also contained cholangiocyte and oligodendrocyte signature genes (Fig. 6b). Cluster 2 (alternative fate cluster) included 3,264 downregulated genes with reduced promoter accessibility and contained eight alternative fate marker genes (Fig. 6b). This bulk analysis mirrored our single-cell experiment with PROX1 decreasing alternative fate signatures, thereby enhancing hepatocyte identity over time (Supplementary Fig. 5). Next, we constructed a target gene regulon of the 4-in-1 reprogramming factors and PROX1 based on DNA-binding and expression data (Supplementary Table 8; Methods). The hepatocyte cluster was enriched for 4-in-1 and depleted for PROX1 targets, while the alternative fate cluster was enriched for direct PROX1 targets (Fig. 6b,c), showing that PROX1 represses alternative fates to promote hepatocyte fate.

### Master regulators *Prrx1* and *Pparg* are repressed by PROX1

**Extended Data Fig. 8:**
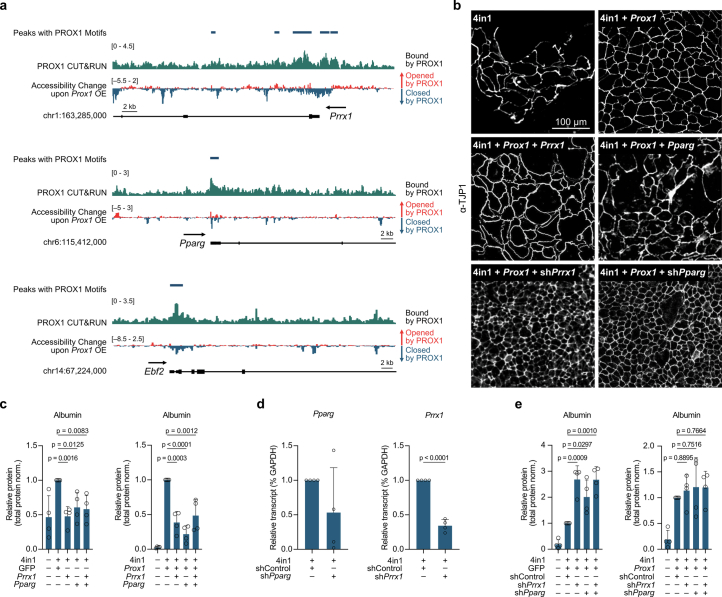
Prrx1 and Pparg are key PROX1 target genes during iHep reprogramming. **a**, IGV tracks of PROX1 CUT&RUN chromatin binding (n = 3) and ATAC-seq accessibility (n = 3) change at day 2 of iHep reprogramming with or without *Prox1* OE at indicated target promoters. **b**, TJP1 immunofluorescence at day 14 of iHep reprogramming with indicated combinations of TF OE and/or shRNA-mediated KD treatments (n = 4). **c**, Albumin western blot levels at day 14 of iHep reprogramming with OE of *Prrx1* or *Pparg* and co-overexpression of GFP or *Prox1* (n = 4). **d**, Expression of *Pparg* and *Prrx1* upon shRNA-KD at day 14 of iHep reprogramming determined by qRT-PCR. **e**, Albumin western blot levels at day 14 of iHep reprogramming upon *Prrx1* or *Pparg* KD with or without *Prox1* OE (n = 4). Scale bar = 100 µm (**b**). Bar graphs show means normalized to total protein levels in **c** and **e** or GAPDH expression (**d**; n = 4), error bars = SD, two-tailed Dunnett’s test (**c,e**) or two-tailed t test (**d**). P values are displayed. Source data

To understand how PROX1 silences nonhepatocyte cell fates, we constructed gene regulatory networks for PROX1 and its direct targets^46^. On day 2, most expression changes were explained by PROX1, while from day 7, we predicted that direct PROX1 targets PRRX1 and EBF2 regulated most effects (Fig. 6d). From day 28, we predicted additional PROX1 targets, including cardiac HAND2 (ref. ^47^), while PROX1 remained important throughout (Fig. 6d). Except for PROX1, all downstream TFs were predicted to act as gene activators, and their target genes were enriched in the repressed alternative fate cluster (Fig. 6b–e). EBF2 is a co-activator of PPARG, and together they can drive adipogenesis^48,49^. PRRX1 is a master TF of stromal fibroblasts^50,51^. We verified that *Prrx1* and *Pparg* were bound and repressed by PROX1 (Extended Data Fig. 8a). Overexpressing *Prrx1* or *Pparg* alone or with *Prox1* impaired liver reprogramming (Fig. 6f,g and Extended Data Fig. 8b,c). Conversely, shRNA-mediated depletion of *Prrx1* or *Pparg* increased hepatocyte conversion only in the absence of *Prox1* OE, indicating that both act downstream of PROX1 (Fig. 6g,h and Extended Data Fig. 8e). Overall, PROX1 suppresses plasticity by repressing master regulators of alternative lineages.

### PROX1 regulates liver cancer plasticity

**Fig. 7:**
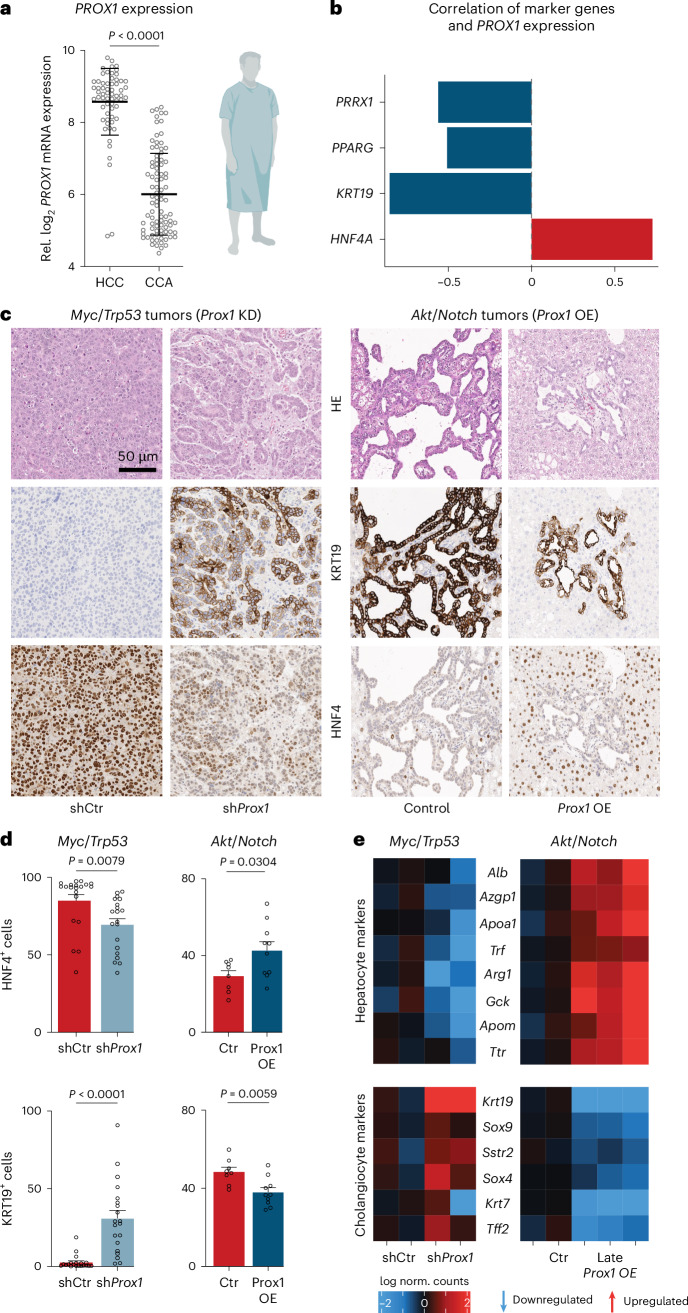
PROX1 regulates HCC versus CCA fate trajectories. **a**, *PROX1* expression in patients with HCC and CCA^24^. The figure is created with BioRender.com. **b**, Pearson correlation of indicated markers with *PROX1* expression in patients from **a**. **c**, Immunohistology of HCC (*Myc*/*Trp53*) and CCA (*Akt*/*Notch*) mice at the endpoint following *Prox1* KD or OE compared to control (*n* = 5). **d**, Quantification of KRT19^+^ and HNF4^+^ cells in GFP^+^ tumors from **c** (*n* = 5, multiple regions per liver). **e**, Selected differentially expressed genes following RNA-seq from mice in (**c**) *Prox1* KD (*n* = 2) or Late *Prox1* OE (*n* = 2–3). Scale bar = 50 µm (**c**). Bar graphs and scatter plots show mean from specified replicates, error bars = s.d., unpaired two-tailed *t* test and *P* values are displayed. HE, hematoxylin and eosin. Source data

**Extended Data Fig. 9:**
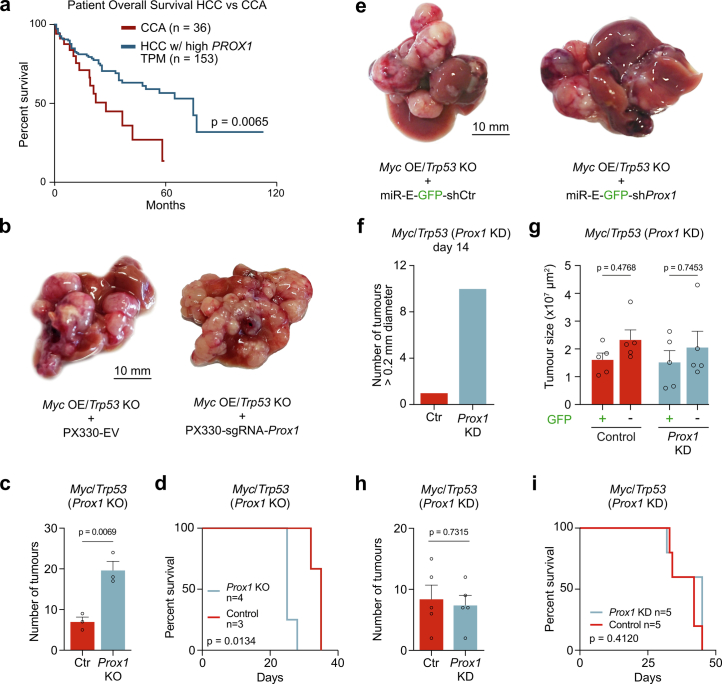
PROX1 loss can enhance HCC liver tumor formation in mice. **a**, Overall survival of patients with HCC and high *PROX1* expression (40% expression cutoff) compared to CCA. **b**, Representative mouse livers at endpoint of HCC induction by *Myc* OE and *Trp53* KO using HDTVI with PX330-sgRNA-*Prox1* KO compared to control (n ≥ 3). **c**, Number of tumors upon treatment as in **b** following *Prox1* KO vs control (Ctr; n = 3). **d**, Survival curve of HCC mice treated as in **b** comparing *Prox1* KO (n = 4) with control (n = 3). **e**, Mouse livers at endpoint of HCC modeling as in **b** with EF1a-GFP-sh*Prox1* KD compared to control (n ≥ 3). **f**, Number of tumor nodules with diameter >0.2 mm treated as in **e** at day 14 (n = 5). **g**, Tumor size (cross-sectional area) treated as in **e** in tumors with or without GFP expression (n = 5). **h**, Tumor numbers upon treatment as in **e** (n = 5). **i**, Survival curve of HCC mice treated as in **e** (n = 5). Scale bar = 10 mm (**b**,**e**). Bar graphs show mean from specified biological replicates, error bars = SD, log-rank test (**a**,**d**,**i**), unpaired two-tailed t test (**c**,**g**,**h**) and Bonferroni correction (**g**). P values are displayed. Source data

Cellular plasticity has a key role in liver cancer; that is, CCA and HCC can both arise from hepatocytes^18,52–54^. However, the factors regulating the transformation of hepatocytes to HCC versus CCA are largely unknown. Strikingly, *PROX1* expression was 1.4-fold higher in HCC than in CCA^24^, and survival in patients with HCC with high *PROX1* expression is better compared to CCA (Fig. 7a and Extended Data Fig. 9a). Intriguingly, the CCA marker *KRT19* and the PROX1 targets *PRRX1* and *PPARG* exhibited a negative correlation with *PROX1* expression in tumor tissue (Fig. 7b), suggesting that PROX1 could regulate liver cancer plasticity and HCC versus CCA fate. Therefore, we performed CRISPR-mediated *Prox1* knockout in our *Myc*/*Trp53* HCC mouse model. This increased tumor numbers greater than twofold and decreased median survival by ~10 days (Extended Data Fig. 9b–d). To follow perturbed cells, we performed *Prox1* knockdown with shRNA constructs coupled to a GFP reporter. This led to a tenfold increase in tumor nodules after 2 weeks without affecting tumor number and survival at the endpoint (Extended Data Fig. 9e–i). Strikingly, *Prox1* knockdown induced a shift from HCC toward CCA, forming glandular tumor structures expressing KRT19 and decreased HNF4 levels (Fig. 7c,d).

**Extended Data Fig. 10:**
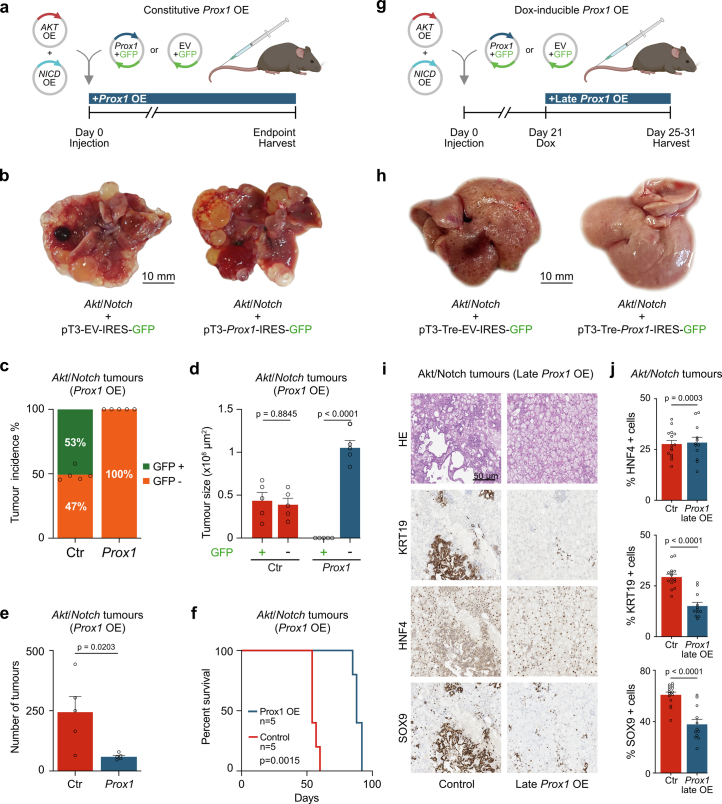
PROX1 can shift liver tumor fate from CCA to HCC in mice. **a**, CCA liver tumor induction via HDTVI-mediated Akt and Notch1 receptor intracellular domain (NICD) (*Akt*/*Notch*) OE with constitutive *Prox1* OE compared to controls. **b**, Mouse livers at endpoint of CCA induction as in **a** (n ≥ 3). **c**, Quantification of GFP^+^ tumors following constitutive OE of *Prox1*-IRES-GFP (n = 4) vs GFP as in **a** (n = 5). **d**, Tumors size (cross-sectional area) in **c** with or without GFP expression indicating transgene expression. **e**, Number of tumors upon treatment as in **c**. **f**, Survival curve of CCA mice treated as in **c**. log-rank test. **g**, Schematic of doxycycline-inducible late *Prox1* OE at day 21 following HDTVI-CCA tumor induction as in **a**. **h**, Mouse livers 1–2 weeks following late *Prox1* OE induced in CCA model as in **g** (n ≥ 3). **i**, Histology of livers treated as in **g**, stained with H&E, KRT19, HNF4 and SOX9 (n = 4). **j**, Quantification of KRT19 and SOX9 (CCA marker) and HNF4 (HCC marker) positive cells in GFP^+^ tumors following late *Prox1* OE (n = 4) vs GFP (n = 4) from **g**. Scale bar = 10 mm and 50 µm (**b**,**h**,**i**). Bar graphs show mean from specified biological replicates, error bars = SD and unpaired two-sided t test. P values are displayed. The figure is created with BioRender.com. Source data

To test whether PROX1 gain affects CCA, we performed HDTVI-mediated overexpression of *Akt* and the Notch1 receptor intracellular domain (*NICD;* *Akt*/*Notch*) in mice^52^. After 2–3 weeks, these mice developed multifocal liver carcinomas with glandular structures, high KRT19 levels and low HNF4α expression (Fig. 7c and Extended Data Fig. 10a,b). Constitutive *Prox1* OE reduced tumor nodules at the endpoint (59.8 versus 245.2 nodules) and increased survival from 54 to 88 days (Extended Data Fig. 10c–f). Strikingly, *Prox1* OE induced a CCA-to-HCC shift, with fewer glandular structures, lower KRT19 expression and higher HNF4 levels (Fig. 7c,d). Inducing *Prox1* OE after 21 days of HDTVI-mediated tumor formation also decreased glandular structures and reduced cholangiocyte marker expression (KRT19 and SOX9; Extended Data Fig. 10g–j). Transcriptome analysis from CCA (*Akt*/*Notch*) and HCC (*Myc*/*Trp53*) mice corroborated these shifts—*Prox1* knockdown in HCC caused downregulation of hepatocyte markers (for example, *Alb* and *Ttr*) and upregulation of cholangiocyte markers (for example, *Krt19* and *Sox9*), while late *Prox1* OE in CCA resulted in the opposite effect (Fig. 7e). In line with *PROX1* expression differences in samples of patients with human liver cancer, manipulating PROX1 in mouse liver cancer models could switch the transformation trajectory of hepatocytes between CCA and HCC. This suggests that PROX1 can maintain hepatocyte cell identity and prevent liver disease in vivo, with lower levels permitting plasticity and higher levels reducing transformation and transdifferentiation.

## Discussion

Many TFs might be expressed in each cell, but only a few so-called master regulators can induce specific lineage identities by gene activation. Their loss, for example, *Pax5* in B cells, can cause plasticity and even cancer^6,7,55,56^. Mounting evidence suggests that repressive TFs also have critical roles in safeguarding cell fate by preventing unwanted plasticity^9^. For example, the repressor Kmg inhibits somatic lineage genes in *Drosophila melanogaster* germ cells^57^. Similarly, the neuron-specific repressor MYT1L suppresses non-neuronal genes in neurons^11,12^. Here we provide evidence that PROX1 represses nonhepatocyte genes to safeguard liver cell identity.

We outline a speculative model for such safeguard repressors. We propose that they regulate genes that are inappropriately accessible^58^ based on (1) affinity to specific DNA motifs while exhibiting (2) cell-type-specific and (3) continuous, lifelong expression. Analysis of 1,296 TFs identified 27 candidates across 18 cell types, with >50% linked to promoting their predicted cell fates. PROX1, essential for hepatocyte commitment^59–61^, meets our safeguard repressor criteria. First, PROX1 exhibited lifelong hepatocyte expression, enhanced reprogramming efficiency greater than tenfold and suppressed alternative fates. Second, *Prox1* deletion reduced hepatocyte reprogramming by ~90% and impaired liver regeneration following injury. Third, PROX1 binding caused decreased chromatin accessibility and repression of alternative fate master regulators.

A repressive role for PROX1 in hepatocytes contrasts its role in other lineages. Notably, PROX1 can function as an activator to promote neurogenesis in the brain^39,40^, or as a master regulator of lymphatic endothelial cells^41,42^. Conversely, in hepatocytes, corepressor interactions are reported^43–45^, and we found that PROX1 interacted with the repressive NuRD complex in the liver but not in the brain. Fusion of the DBD of PROX1 to activator and repressor domains confirmed that PROX1 target gene repression promoted hepatic fate, while target gene activation induced alternative fates. Hence, depending on cofactor interactions, PROX1, like other TFs, can switch between activation and repression. Further studies will show whether other TFs guard cell fate in this dual, cofactor-dependent manner.

We found decreased *PROX1* expression in patients with liver cancer, which supports the role of cell fate plasticity in disease. In mice, *Prox1* OE reduced neoplastic transformation and progression across three liver cancer models. Strikingly, median survival almost doubled in our models and increased by 2–3 weeks, double the increase induced by the kinase inhibitor sorafenib, a standard of care in unresectable HCC, in comparable models^62^. This contrasts studies where *Prox1* OE in tumor cells induced migratory and metastatic potential upon transplantation^22,63^. Supporting a role in suppressing tumor initiation, *Prox1* loss accelerated tumor formation in our HCC model. Strikingly, resulting tumors displayed an identity switch from HCC toward CCA. Conversely, in patients with CCA, *PROX1* levels are low, and its OE in mouse models was able to shift CCA to HCC-like tumors. Indeed, hepatocytes can give rise to both HCC and CCA^18^, and our data suggest that PROX1 regulates this decision. Interestingly, in the context of colorectal cancer, PROX1 has been recently described as suppressing lineage plasticity by repressing nonintestinal genes^64^, supporting the notion that it can prevent cell fate plasticity and cancer.

In conclusion, we show that continuous cell-type-specific repression of alternative fates is essential for cell fate induction and maintenance. Identifying and mechanistically characterizing similar factors, guided by computational tools such as the one presented here, will be essential to test whether this concept extends to other cell types, could help generate cells for biomedical applications and reveal targets that prevent cell fate plasticity and disease.

## Methods

### Human material

Formalin-fixed, paraffin-embedded human liver tissue samples were retrieved from the Medical Faculty Mannheim, Heidelberg University, for immunohistology analyses. Specimens were collected with informed patient consent in accordance with the approval by the Institutional Review Board of the University Hospital Mannheim (permit 2012-293N-MA).

### Primary mouse cell lines

MEFs were collected from E13.5 embryos of C57BL/6N (wild type) or C57BL/6J (*Prox1*^*fl*/fl^) mice^36^ as described^12,65^. The distal portions of all limbs from three to four embryos were dissected, placed in 100 μl trypsin, cut thoroughly and incubated in 1 ml trypsin (at 37 °C for 15 min). Trypsin was inactivated by the addition of cell suspension to 25 ml MEF media (DMEM; Invitrogen) containing 10% cosmic calf serum (Hyclone), β-mercaptoethanol (Sigma), nonessential amino acids, sodium pyruvate, l-glutamine and penicillin–streptomycin (all from Invitrogen). MEFs were then cultured at 37 °C with 5% CO_2_ in MEF media and either cryopreserved or passaged twice using trypsin before reprogramming experiments.

### HCC cell lines

Mouse primary liver cancer cell lines were derived from C57BL/6N female mice following HDTVI with either *Myc* OE/*Trp53* KO or *Kras*(G12D) OE/*Trp53* KO^33^. Mouse primary liver cancer and human Hep3B cells were transduced with lentivirus prepared from indicated plasmids (Supplementary Table 10) in DMEM (Invitrogen) containing 10% fetal bovine serum (Sigma), nonessential amino acids, sodium pyruvate, l-glutamine and penicillin–streptomycin (all from Invitrogen). Puromycin selection was performed (2 μg ml^−1^) for 2 days to generate stable cell lines. Cellular proliferation rates were determined beginning 1 day after seeding with media containing 2 μg ml^−1^ or the indicated amount of doxycycline (Sigma) or without doxycycline (for controls) for a total of 2–10 days (depending on the proliferation rate of the line) using the IncuCyte S3 live-cell imaging system (Sartorius). Four brightfield images per well were acquired every 4 h at a magnification of ×10 and analyzed using IncuCyte S3 2019B software. Experiments were performed at 37 °C with 5% CO_2_ in two to three biological replicates, with five to six technical replicates each.

### Animal experiments

For HDTVI, 2 ml of sterile 0.9% NaCl solution, corresponding to 10% of body weight, containing the plasmids of interest, was injected into the tail vein of 8-week-old female C57BL/6N mice within 5–7 s^19,33,66–68^. Depending on the vector used, this technique allowed for liver-specific gene knockouts and/or overexpression by in vivo transfection of hepatocytes. Each mouse was injected with 20 µg of pX330-based plasmid for sgRNA-mediated gene knockout of *Tp53* or *Prox1* and 10 µg of pT3-EF1a-based plasmid for transposon-mediated stable overexpression of *Myc, Kras, Akt or NICD* with or without rtTA. For *Prox1* knockdown, 2 µg of CMV-Sleeping Beauty transposase and 20 µg of miR-E-based plasmid with *Prox1*- or Renilla Luciferase-targeting shRNA and a GFP reporter were co-injected. For constitutive *Prox1* OE, 4 µg of CMV-Sleeping Beauty transposase and 10 µg of pT3-PGK-based or pT3-EF1a-based plasmid with or without *Prox1*-cDNA and a GFP reporter were co-injected (Supplementary Tables 6 and 10). For late *Prox1* OE, 4 µg of CMV-Sleeping Beauty transposase and 10 µg of pT3-Tre-based plasmid with or without *Prox1*-cDNA and a GFP reporter were co-injected. Each experimental group involving HDTVI contained at least five mice, with all mice monitored daily. For mice that received Tre-driven transgenes, a doxycycline-containing diet (6.25% doxycycline hyclate; Envigo Teklad) was given beginning at day 14 or 21 post-HDTVI, depending on the model. Upon killing of mice (at indicated time points or at humane endpoints), relevant organs were collected and photographed. Survival data were analyzed based on the time between HDTVI and killing at a humane endpoint. After killing, tumor samples were taken for RNA and protein analysis. The remaining tissue was incubated in 4% paraformaldehyde for a minimum of 24 h for subsequent histological analysis. For DDC-induced liver injury, 4-month-old C57BL/6J *Prox1*^*fl*/*fl*^ mice^36^ were injected into the tail vein with 150 µl of PBS with 5 × 10^11^ genomic particles of adeno-associated virus 8 (AAV8) carrying *Cre* or Δ*Cre*-recombinase (Supplementary Table 10). After 14 days, liver injury was induced by providing 0.1% DDC (Sigma, 137030) mixed with a standard diet (KLIBA NAFAG, 3437) for 2 weeks, followed by a normal diet for another 2 weeks. After killing, blood and tissue were collected for subsequent analysis. All animal experiments complied with ethical regulations and were approved by the regional ethics board in Karlsruhe, Germany.

### Blood biochemical analysis

Blood was collected, and serum was freshly isolated by centrifugation (12,000*g* for 10 min at 4 °C). Serum was stored at −20 °C until analysis. Aspartate transaminase, ALP and alanine aminotransferase levels were detected as per manufacturer instructions (Fujifilm DRI-CHEM SLIDE).

### Histology

Paraformaldehyde-incubated livers were embedded in paraffin, and 2 μm slices were processed for automated immunohistochemistry staining using BOND-MAX (Leica Biosystems). Bond citrate solution (Leica Biosystems, AR9961), Bond EDTA solution (Leica Biosystems, AR9640) or Bond proteolytic enzyme kit (Leica Biosystems, AR9551) were used for antigen retrieval (Supplementary Table 11). Sections were incubated in antibodies diluted in Bond primary antibody diluent (Leica Biosystems, AR9352) followed by secondary antibody (Leica Biosystems) incubation and staining with the Bond Polymer Refine Detection Kit (Leica Biosystems, DS9800). Slides were scanned with an Aperio AT2 slide scanner (Leica Biosystems) at ×20, then annotated and analyzed with Aperio ImageScope (v12.4.0.5043; Leica Biosystems) to determine the size of tumor nodules. Marker staining quantifications were analyzed in QuPath (v0.4.3)^69^ using the positive cell detection option with the same settings for each marker across all sections. If possible, all or multiple regions were analyzed per liver section. Certified pathologists (H.W. and D.T.) performed the histopathological analysis of paraffin-embedded liver tumor sections.

### PROX1 immunoprecipitation and liquid chromatography–tandem mass spectrometry (LC–MS/MS) analysis

For each immunoprecipitation, one hippocampus or liver of 2–3-month-old C57BL/6N mice was used per biological replicate. The fresh tissue was lysed in 1 ml lysis buffer containing (in mM) 0.5% Tween-20, 50 Tris (pH 7.5), 2 EDTA, 1 DTT, 1 PMSF, 5 NaF (all from Sigma) and complete protease inhibitor (Roche) for 15 min at 4 °C and processed for immunoprecipitation using 2 μg PROX1 or control IgG (Sigma) antibody per reaction (Supplementary Table 11)^12,14^. After elution, bound proteins were enzymatically digested with trypsin using an AssayMAP Bravo liquid handling system (Agilent Technologies) running the autoSP3 protocol as described here^70^. An LC–MS/MS analysis was carried out using a Vanquish Neo UPLC system (Thermo Fisher Scientific) directly connected to an Orbitrap Exploris 480 mass spectrometer for a total of 60 min per sample. Peptides were online desalted on a trapping cartridge (Acclaim PepMap300 C18; 5 µm, 300 Å wide pore; Thermo Fisher Scientific) with a loading volume of 60 µl using 30 µl min^−1^ flow of 0.05% Trifluoroacetic acid (TFA) in water. The analytical multistep gradient (300 nl min^−1^) was performed with a nanoEase MZ Peptide analytical column (300 Å, 1.7 µm, 75 µm × 200 mm; Waters) using solvent A (0.1% formic acid in water) and solvent B (0.1% formic acid in acetonitrile). For 45 min, the concentration of B was linearly ramped from 5% to 30%, followed by a quick ramp to 80%. After 4 min, the concentration of B was lowered to 2%, and a three-column volume equilibration was appended. Eluting peptides were analyzed in the mass spectrometer using data-dependent acquisition mode. A full scan at 60k resolution (380–1400 *m*/*z*, 500% AGC target and 100 ms maxIT) was followed by up to 1.5 s of MS/MS scans. Peptide features were isolated with a window of 1.2 *m*/*z*, fragmented using 26% NCE. Fragment spectra were recorded at 15k resolution (100% AGC target and 150 ms maxIT). Dynamic exclusion was set to 10 s. Each sample was followed by a wash injection to avoid carryover. System readiness was assessed before, during and after the measurements via an in-house quality control (QC) pipeline. Data analysis was carried out by MaxQuant (v.2.1.4.0)^71^ using an organism-specific database extracted from Uniprot.org (mouse reference database with one protein sequence per gene, containing 21,957 unique entries from 3 May 2023). Settings were set to default with the following adaptations. Separate parameter groups were assigned for liver and hippocampus samples. Separate label-free quantification (LFQ) per parameter group was enabled. Besides the LFQ approach based on the MaxLFQ algorithm^72^, quantification was also done using intensity-based absolute quantification (iBAQ) values^73^. The statistical analysis of proteins has been conducted as follows: adapted from the Perseus recommendations^74^, protein groups with valid values in 70% of the samples of at least one condition were used for statistics. In addition, missing values, being completely absent in one condition, were imputed with random values drawn from a downshifted (2.2 s.d.) and narrowed (0.3 s.d.) intensity distribution of the individual samples. For missing values with no complete absence in one condition, the R package missForest (v.1.5)^75^ was used for imputation. No additional normalization was applied to the iBAQ values that were used in the statistical analysis. The statistical analysis was performed with the R package limma (v.3.54.0)^76^ with an adapted contrast setup from Ch. 9.5 Interaction Models. Within the eBayes function, the options robust and trend were set to TRUE. The *P* values were adjusted with the Benjamini–Hochberg method for the multiple testing. PROX1 interactors were considered significant with an absolute log(fold change (FC)) > 1, *P* value < 0.05 and quality score > 0.5. In addition, interactors were filtered according to nuclear location (based on https://www.proteinatlas.org/about/download). The mass spectrometry data analysis can be found in Supplementary Table 9.

### Recombinant virus production

Lentivirus was produced through transfection of lentiviral backbones containing indicated transgenes along with third-generation packaging plasmids into HEK293T cells according to the Trono laboratory protocol (Supplementary Table 10)^77^. Lentivirus was concentrated from HEK293T culture supernatant through ultracentrifugation (69,000*g* for 2 h at 4 °C) and stored at −80 °C or used immediately. AAV8 vectors were produced by polyethyleneimine triple transfection of HEK293T cells using indicated plasmids (Supplementary Table 10)^78^. AAV vectors were purified using iodixanol gradient density centrifugation followed by buffer exchange to PBS. AAV vector quantification was conducted by droplet digital (dd)PCR using the Bio-Rad ddPCR system. Each 20 µl PCR contained 5 µl diluted virus template, 10 µl of the ddPCR Supermix for Probes (no dUTP; Bio-Rad), 4 µl nuclease-free H_2_O and 1 µl inverted terminal repeat (ITR)-primer/probe mix (final concentration—900 nM for primers and 250 nM for probes; ITR_f: GGAACCCCTAGTGATGGAGTT, ITR_r: CGGCCTCAGTGAGCGA and ITR_probe: HEX-CACTCCCTCTCTGCGCGCTCG-BHQ1). The measured copy number of vector templates per reaction was corrected by the input volume and dilution factor to calculate vector genomes per microlitre vector stock.

### Direct reprogramming from MEFs

Wild-type C57BL/6N or *Prox1*^*fl*/*fl*^ C57BL/6J MEFs were transduced by incubation with lentivirus prepared from indicated plasmids (Supplementary Table 10) in MEF medium with 8 μg ml^−1^ polybrene (Sigma) for 16–20 h. Medium was exchanged to MEF medium containing 2 μg ml^−1^ doxycycline (Sigma) to induce transgene expression. For myocyte and neuronal reprogramming, MEFs were transduced with lentivirus containing rtTA and *Myod1* or *Ascl1* (refs. ^11,12^), respectively, along with the indicated lentivirus. After 48 h, all medium was exchanged with N3 medium (DMEM/F12) containing N2 supplement, B27, 20 μg ml^−1^ insulin, penicillin–streptomycin (all from Invitrogen) and doxycycline to continue transgene expression. The medium was changed every 2 days for the remainder of the reprogramming. For hepatocyte reprogramming^20^, MEFs were seeded onto collagen-coated plates and transduced 1 day later with 4-in-1 and rtTA lentivirus, together with the indicated lentivirus as above. For CUT&RUN and ATAC–seq at day 2, transduction was performed in MEF medium containing 2 μg ml^−1^ doxycycline (Sigma) to induce transgene expression, and cells were collected after one media exchange at day 2. For long-term reprogramming, 1 day following doxycycline induction, the medium was supplemented with 0.5× volume hepatocyte culture medium (HCM; Lonza) containing 5% FBS (Life Technologies) and 2 μg ml^−1^ doxycycline. On day 2, all medium was exchanged to a mixture of 1/3 MEF medium and 2/3 HCM + 5% FBS with 2 μg ml^−1^ doxycycline. On day 3, all medium was exchanged to HCM + 5% FBS with 2 μg ml^−1^ doxycycline. Medium was then changed every 2 days for the remainder of reprogramming. For domain fusion experiments, we followed published protocols^65,79^, and for shRNA-based knockdown, we treated cells with lentivirus targeting the indicated gene with two independent shRNA constructs (Supplementary Table 10). All cells were cultured at 37 °C with 5% CO_2_.

### Immunofluorescence quantification

To calculate the efficiency of neuronal induction, the total number of TUBB3-expressing cells with complex neurite outgrowth (cells with a round cell body and at least one thin process with a length at least double the diameter of the cell body) was counted manually^12^. Any TUBB3-positive and desmin-negative cells that did not meet the morphological criterion were considered TUBB3-positive, non-neuronal cells (Extended Data Fig. 7f). To calculate the efficiency of myocyte cell induction, the total number of desmin-expressing cells was counted manually. Dual TUBB3- and desmin-positive cells were considered as a separate category of mixed identity cells. To calculate the efficiency of hepatocyte reprogramming, the total number of cells for which TJP1 staining formed a complete border around the nucleus (stained by DAPI) was counted manually. All quantifications were performed 14 days after transgene induction by immunofluorescence microscopy. Fluorescence micrographs were captured automatically using a Nikon Ti2 microscope with a Ti-HCS system, the Nikon S Plan Fluor ELWD ×20 Numerical Aperture (NA) 0.45 objective, the Nikon DS-Qi2 CMOS camera (2,404 × 2,404) and the Lumencor Sola SE II light source. Quantifications were based on the mean number of positive cells across five to ten randomly selected ×20 magnification fields of view per biological replicate, with at least three biological replicates. The number of reprogrammed cells in each treatment condition was then normalized to the number of reprogrammed cells in the control condition. To calculate the fraction of total reprogrammed cells positive for indicated markers and morphological criteria, the number of cells in each of the five categories was divided by the total number of cells present in any of the five categories, pooled across all replicates.

### Computational safeguard repressor screen

Using single-cell gene expression and cell-type annotations from Tabula Muris^16^, we analyzed the expression of 1,296 TFs^80^ in 18 selected cell types using median normalized CPM units. In addition, we retrieved the top 1,000 cell-type-enriched genes using Seurat FindMarkers() (Supplementary Table 1). In the promoters of these signature genes (±2 kb around the TSS), we determined the number of DNA-binding motifs for each TF (derived from CIS-BP 1.94d^80,81^ mapped to mm10). Pairwise comparisons were performed between cell-type-specific gene signatures to remove overlapping genes and calculate mean TF motif density. For each cell type, TF expression specificity and TF-binding motif enrichment in signature gene promoters were calculated by *z-*score scaling to obtain *Z*_expression_ and *Z*_motif_, respectively. The higher *Z*_expression_, the more specifically the TF is expressed in the corresponding cell type. TFs with positive *Z*_motif_ and high *Z*_expression_ might function as activators of cell-type-specific genes. Conversely, a negative *Z*_motif_ indicates depletion of DNA-binding motifs of this TF at genes specific to the analyzed cell type, indicating that it could act as a safeguard repressor by silencing cell-type-unspecific genes. To compare motif-based prediction with actual TF chromatin binding, we used our PROX1 primary liver CUT&RUN peaks filtered by PROX1 motifs from the Hocomoco v12 motif database^82^ and selected peaks within ±2 kb distance to any TSS. These gene-associated PROX1 peaks were tested for enrichment at cell-type marker gene sets obtained from Panglao using one-tailed hypergeometric tests, which showed significant enrichment at several nonhepatocyte signature genes, such as fibroblasts. We deployed a searchable database of this bioinformatics analysis at https://apps.embl.de/safeguard/. In addition, we calculated a safeguard repressor score for each TF defined as the sum of *Z*_expression_ and −1 × *Z*_motif_, following *Z*_max_ normalization to ensure equal weighting of expression and motif bias (Fig. 1b, Extended Data Fig. 1c and Supplementary Table 2). TFs were further annotated as cell-type-specific and lifelong expressed using data from Tabula Muris Senis^16^ based on the following two criteria: (1) the mean expression in Tabula Muris Senis must be at least 50% of that in Tabula Muris, and (2) the mean expression must be higher in a specific cell type compared to the mean expression across all 18 cell types in Tabula Muris. In addition, we categorized TFs as lifelong expressed using a mouse developmental gene expression atlas^17^ for the brain, heart and liver if they exhibited high expression (in the highest quantile) in a continuous manner (in more than 70% of developmental time points and replicates). We analyzed safeguard candidates for microglia, neurons, oligodendrocytes and astrocytes from the brain data; cardiomyocytes from the heart data; and hepatocytes from the liver data. In total, 77% of the safeguard repressor candidates in these cell types (17/22), including *Prox1* and *Myt1l*, were found to be lifelong expressed in an organ-specific manner, which, compared to all TFs expressed in these tissues, reached statistically significant enrichment using a Fisher’s exact test. Top safeguard repressor candidates were also categorized as activators, repressors or dual activator/repressors, as well as having a known tumor suppressor role or not, in the cell type of interest, based on the literature review (Supplementary Table 2).

### Bulk RNA-seq library generation

Primary mouse liver tumor samples were dissected and placed into TRIzol (Invitrogen). Samples were crushed and then homogenized using QIAshredder (Qiagen). Reprogrammed hepatocytes, or Hep3B cells, were collected from culture plates by adding TRIzol to the cultures at indicated time points. RNA collected in TRIzol was isolated using the RNA Miniprep Kit (Zymo Research). For bulk RNA sequencing, libraries were prepared according to the dUTP protocol^83^ and paired-end sequencing (2 × 100 bp) was performed on the NovaSeq 6000 (Illumina).

### RNA-seq data processing

Raw reads were mapped to the reference genome mm10 or hg38 using STAR^84^. Differential gene expression was determined using DESeq2 (R package v.1.28.1)^85^ with size factor normalization and Wald significance tests. For bulk MEF reprogramming data, we used the primary MEF line as a covariate. ComplexHeatmap (v.2.12.1)^86^ was used to generate heatmaps. In Fig. 6a, genes were included in the analysis if they had abs(log_2_(FC)) > 0.75 at any time point.

### CUT&RUN library preparation

Cells were collected with Accutase and strained through a 70 μm strainer followed by CUT&RUN processing^87^. In total, 250,000 cells were washed twice with 1 ml wash buffer (20 mM HEPES–KOH (pH 7.5), 150 mM NaCl, 0.5 mM spermidine and 1× Roche Complete Protease Inhibitor) and then resuspended in 200 μl of ice-cold cell lysis buffer (10 mM Tris–HCl (pH 7.5), 10 mM NaCl, 3 mM MgCl_2_, 0.1% Tween-20, 0.1% NP-40 and 1% BSA in ddH2O) for 3–5 min. In total, 1 ml of ice-cold wash buffer was then added to stop cell lysis, and nuclei were centrifuged (500*g* for 10 min at 4 °C). Nuclei were resuspended in 200 μl wash buffer, and 100,000–150,000 nuclei were taken to another tube. Concanavalin-A beads (Polysciences) were pre-activated in cold binding buffer (20 mM HEPES–KOH (pH 7.5), 10 mM KCl, 1 mM CaCl_2_ and 1 mM MnCl_2_). Nuclei were centrifuged, and the buffer was removed. Activated beads were then added to the pellet. The bead–cell suspension was rotated (at room temperature for 10 min). The supernatant was removed on a magnet, and the beads were resuspended in antibody buffer (0.2 mM EDTA, 0.05% wt/vol digitonin in wash buffer). In total, 1 µg primary antibody (rabbit anti-FLAG, in vitro, or rabbit anti-Prox1, in vivo) or control (rabbit IgG; Sigma) was added (Supplementary Table 11), and cells were incubated on a nutator (overnight, 4 °C). Beads were washed twice in digitonin-wash buffer (0.05% wt/vol digitonin in wash buffer), resuspended in 700 ng ml^−1^ pAG-MNase (Protein Expression and Purification Core Facility, EMBL) in digitonin-wash buffer and rotated (at 4 °C for 1 h). pAG-MNase-loaded beads were then washed twice in a digitonin-wash buffer, resuspended in a digitonin-wash buffer and placed on ice. In total, 1 µl of 100 mM CaCl_2_ was added to induce chromatin digestion, and the mixture was incubated on ice (30 min). In total, 50 µl of 2× stop buffer (340 mM NaCl, 20 mM EDTA, 4 mM EGTA, 0.05% wt/vol digitonin, 50 µg ml^−1^ RNase A, 50 µg ml^−1^ glycogen and 0.5 ng ml^−1^ spike-in *Escherichia coli* DNA) was added, and the suspension was incubated at 37 °C for 10 min to release chromatin fragments from cells. The supernatant was subjected to phenol–chloroform extraction, and purified DNA fragments were used for library preparation with the NEBNext DNA Library Prep Kit for Illumina (NEB, E7645). Libraries were then sequenced (paired-end, 2 × 40 bp) on the NextSeq 550 and 2000 platform (Illumina). Livers from 2- to 3-month-old C57BL/6J mice were collected and directly processed for nuclei isolation using liver swelling buffer (10 mM Tris (pH 7.5), 2 mM MgCl_2_ and 3 mM CaCl_2_) with the help of a douncer. Homogenized tissue was passed through a 70 µm strainer and centrifuged (at 400*g* for 5 min at 4 °C). Tissue pellets were resuspended in liver lysis buffer (10 mM Tris (pH 7.5), 1% NP-40, 2 mM MgCl_2_, 10% glycerol and 3 mM CaCl_2_) and centrifuged again. Pellets containing the nuclei were washed twice in PBS, and then the same protocol as for cells was followed. Then, the samples were processed as described for cells. For mouse livers, 500,000 nuclei were used per sample instead of 100,000–150,000 for cells. In addition, 5 μg of target antibody was used per sample.

### CUT&RUN data processing

CUT&RUN data were analyzed using the nf-core/cutandrun pipeline (v1.0.0) with Nextflow (v21.05.0)^88,89^. Reads were aligned to mm10 or hg38. Software versions used were as follows: BEDtools (v2.30.0)^90^, Bowtie 2 (v2.4.2)^91^, deepTools (v3.5.0)^92^, DESeq2 (v1.28.0)^85^, FastQC (v0.11.9), MultiQC (v1.11)^93^, Picard (v2.23.9)^94^, Python (v3.8.3), SAMtools (v1.10)^95^, Genrich (v0.6.1; https://github.com/jsh58/Genrich), TrimGalore (v0.6.6)^96^ and UCSC (v377). Consensus peaks were defined by running Genrich -m 30 -e chrM -r -l 5 -q 0.3. To determine peaks containing motifs, we mapped the PROX1 binding motif (CIS-BP ID: M03445_2.00) onto mm10 or hg38 using the scanMotifGenomeWide.pl function in Homer (v4.11), extended each resulting motif peak to a total width of 50 bp and ran bedtools intersect -wa on the CUT&RUN consensus peaks and motif peaks, respectively. Genes defined as direct target genes based upon CUT&RUN were determined by running Homer annotatePeaks.pl on the final peak set and filtering for genes with a peak within ±1 kb of their TSS. An overview of the CUT&RUN experiments is in Supplementary Table 13, and the final motif-containing peaksets for liver, Hep3B and induced hepatocytes are in Supplementary Table 3.

### ATAC–seq library preparation

Cells on culture plates were washed twice with PBS and detached by Accutase digestion (4–6 min at room temperature) followed by ATAC processing. Cell suspensions were placed into an equal volume of MEF medium followed by centrifugation (500*g* for 5 min at 4 °C) before resuspension in ice-cold PBS + 1% BSA. Cells were then strained through a 70 μm filter and centrifuged (500*g* for 5 min at 4 °C). In total, 50,000 cells or nuclei were resuspended in 50 μl of ice-cold cell lysis buffer containing 0.1% NP-40 and 0.01% digitonin in wash buffer (10 mM Tris–HCl (pH 7.5), 10 mM NaCl, 3 mM MgCl_2_, 0.1% Tween-20 and 1% BSA in ddH_2_O) for 3–5 min. In total, 1 ml of ice-cold wash buffer was then added to stop cell lysis, and nuclei were centrifuged (500*g* for 10 min at 4 °C). In total, 4.5 μl of each of the tagmentation oligos, Tn5_ME and Tn5-R1N (Supplementary Table 12), were annealed in oligo annealing buffer (10 mM Tris–HCl (pH 7.5), 50 mM NaCl and 10 mM EDTA final concentration in ddH_2_O) by heating at 95 °C (3 min) followed by a ramp down by 1–25 °C. The same procedure was performed for Tn5_ME and Tn5-R2N (Supplementary Table 12). Tn5 was assembled with annealed oligos by combining 50 μl Tn5 (1 mg ml^−1^ stock) with 25 μl annealed Tn5_ME and Tn5-R1N and 25 μl Tn5_ME and Tn5-R2N. Nuclei were resuspended in 40 μl tagmentation buffer (38.8 mM Tris acetate, 77.6 mM K-acetate, 11.8 Mg acetate, 18.8% dimethylformamide and 0.12% NP-40 in ddH_2_O), to which 5 μl of ice-cold PBS + 1% BSA and 5 μl of pre-assembled Tn5 was added. Samples were incubated on a Thermomixer (37 °C for 30 min at 500 rpm) before being subjected to MinElute (Qiagen) cleanup to extract tagmented DNA. Eluted DNA was pre-amplified with P5 and P7 primers (Supplementary Table 12) using the NEBNext HF 2× PCR Master Mix (New England Biolabs) in a thermocycler set to 72 °C (5 min), 98 °C (30 s) and five cycles of 98 °C (10 s), 63 °C (30 s) and 72 °C (1 min). A qPCR side reaction was performed with the resulting pre-amplified libraries to determine the necessary additional cycles (five cycles fewer than the number of cycles corresponding to 1/3 of max fluorescence) for complete amplification. After finishing amplification, 50 μl of each library was subjected to two-sided size selection by the addition of 27.5 μl (0.55×) AMPure XP beads, the incubation (5 min), transfer of supernatant to new tubes, the addition of 42.5 μl (1.4×) AMPure XP beads to the supernatant, the incubation (5 min), three washes with 80% ethanol and elution of DNA. The resulting libraries were then sequenced on the NextSeq 2000 platform (Illumina).

### ATAC–seq data processing

ATAC–seq data were analyzed with a custom Snakemake pipeline. Raw reads were quality-checked with FastQC (v0.11.8), trimmed with trimmomatic (v0.38) and aligned to UCSC mm10 or hg38 with Bowtie 2 (v2.3.4.3)^91^. Aligned reads were cleaned and base-recalibrated (to take account of Tn5 insertion biases) with SAMtools (v1.10)^95^ and Picard (v2.18.16)^94^. Reads were filtered with BEDtools (v2.27.1)^90^, SAMtools and Picard. Peaks were called using MACS2 (2.1.2), and coverage was calculated with deepTools (v3.1.3)^92^. Final quality checks were performed with MultiQC (v1.6)^93^. Differential peak analysis was performed with DiffBind (v3.4.11)^97^ as described in the authors’ vignette (same version). For the enrichment analysis, we analyzed significant differentially accessible regions (adjusted *P* < 0.05, log_2_(FC) < 0) using the entire ATAC–seq peak set as a background for the Genomic Regions Enrichment of Annotations Tool (v4.0.4)^98^.

### Footprint analysis

To determine the PROX1 footprint, 11 bp-wide motif peaks (CIS-BP ID: M03445_2.00) were mapped on mm10 using the scanMotifGenomeWide.pl function in Homer (v4.11) and intersected with CUT&RUN consensus peaks using BEDtools intersect -wa. These generated peaks are present in the CUT&RUN consensus peak set that contains motifs and are centered on the motif. DiffTF (v1.8)^99^ was then run using ATAC–seq BAM files and the motif-centered peak set^99^.

### Single-cell RNA-seq multiplexing and library generation

For single-cell RNA-seq, reprogrammed cells were dissociated into single cells after two PBS washes by Accutase digestion. Cells were lifted using cell scrapers, and suspensions were then passed through 70 μm filters into 9 ml of prewarmed MEF medium. Cells were pelleted (800*g* for 5 min) and resuspended in 1 ml HBSS + 0.04% BSA to >3.5 million cells per ml and then fixed through the addition of 4× volume of ice-cold methanol to a final concentration of 80% methanol. Cells were then stored at −20 °C. Barcoding oligonucleotides for multiplexing were designed based on the ClickTag scheme^100^ and ordered with a 5′ amine group label (Supplementary Table 12). A methyltetrazine group was conjugated to the oligos via the 5′-amine group. Membrane proteins on the fixed cells were conjugated to an amine-*trans*-cyclooctene group. Subsequent incubation of oligos and cells according to the ClickTag protocol^100^ allowed chemical labeling of cells. Approximately 10,000 cells were multiplexed and loaded per gel beads-in-emulsion (GEM) well in the Chromium Controller (10x Genomics), and the Chromium Single Cell 3′ v2 reagent kit was used according to the manufacturer’s instructions for gene expression library generation. We performed modifications at the cDNA and library preparation steps as suggested in the ClickTag protocol to generate barcoded oligonucleotide libraries in parallel. Gene expression and barcode libraries were then diluted to equimolar amounts and pooled at a 9 to 1 ratio, and 26 + 98 bp paired-end sequencing was performed using the NovaSeq 6000 (Illumina).

### Single-cell RNA-seq data processing

Reprogramming single-cell RNA-seq data generated in this study were analyzed using 10x Genomics Cell Ranger (v4.0.0) and Seurat (v4.0 and v4.3)^101^. Cells containing fewer than 1,000 features, containing fewer than 2,000 reads or with mitochondrial genes comprising over 20% of genes were discarded. Cell doublets were removed using Scrublet^102^ with a threshold of 0.35. Cells were demultiplexed based on their hashtag oligos by running Seurat HTODemux() recursively. Expression values of cell cycle genes were regressed out using vars.to.regress in ScaleData() to reduce heterogeneity caused by cycling cells. Cells were projected into two-dimensional space using the Uniform Manifold Approximation and Projection (UMAP) algorithm. Cells were clustered using the Louvain clustering algorithm with 40 dimensions and a resolution parameter of 0.35.

### Regulon analysis

The PROX1 regulon was defined as genes that exhibited transcriptional downregulation in PROX1 repressor fusion or upregulation in activator fusion and contained a PROX1 CUT&RUN peak and motif within ±1 kb of the TSS. For all other TFs, Dorothea (v1.7.2, all confidence levels)^103^ was used to build their target gene regulon. Genes in each regulon can be found in Supplementary Table 8. We constructed a subgene-regulatory network containing the PROX1 regulon and the regulons of TFs that are directly regulated by PROX1. GRaNPA was used to determine the most important TFs based on this gene regulatory network and differential expression analysis between *Prox1* OE and control^46^.

### Activity score quantification for 4-in-1 TFs and Prox1

The 4-in-1 TFs activity was inferred based on the aggregate expression of all genes within the 4-in-1 regulon from Dorothea, calculated using Seurat AddModuleScore(). PROX1 activity was calculated by taking the inverse of the aggregate expression, calculated with AddModuleScore(), of a subset of the PROX1 regulon that consists of 79 high-confidence PROX1-repressed target genes (Supplementary Table 8). These contained a PROX1 CUT&RUN peak and motif within ±1 kb of the TSS, and their promoters were differentially closed at day 2 following PROX1 OE based on the ATAC–seq log_2_(FC) threshold (GFP versus *Prox1 OE*) of >1.

### Filtering cells without transduction

We used PROX1 activity as a basis for the removal of cells from the Prox1 condition that were not successfully transduced with *Prox1* OE lentivirus. For each Prox1-labeled cell, we determined the proportion of GFP-labeled cells with a lower PROX1 activity than that cell (GFP_proportion_) and the proportion of Prox1-labeled cells with a higher PROX1 activity than that cell (Prox1_proportion_). We then excluded cells in which GFP_proportion_ was larger than Prox1_proportion_. A similar process was used in 4-in-1- and MEF-labeled cells to remove cells from the dataset that were not transduced with 4-in-1 OE lentivirus.

### Signature gene analysis

For analysis of cell-type gene signatures, marker genes from the Panglao database^104^ were used as input with Seurat AddModuleScore(). The Pearson correlation coefficient between cell-type geneset scores and 4-in-1 or PROX1 activity across all cells was used as the correlation score between cell identity and TF activity.

### Re-analysis of Myc-driven liver tumor and DDC-liver injury scRNA-seq data

Published Myc-driven liver tumor single-cell RNA-seq^34^ data were re-analyzed using Seurat and the provided metadata. After data normalization with SCTransform, we performed dimensional reduction using PCA and UMAP, using the top 30 dimensions. Clustering was performed using the Louvain clustering algorithm with a resolution of 0.2. To ascertain cell identity, we calculated scores based on the Panglao dataset using Seurat module scores. For our specific study objectives, we narrowed our focus to wild-type samples classified as ‘healthy’ and those from day 28 upon Myc OE. Published DDC-liver injury single-cell RNA-seq data^35^ were re-analyzed using Seurat in conjunction with provided metadata, focusing on cells generated using the 10× platform and samples from DDC-injected mice. After data normalization with SCTransform, dimensional reduction was done using UMAP and PCA based on 50 dimensions. We used predefined cell-type annotations by the authors and calculated cell identity using Seurat module scores with reference to the Panglao dataset. Pseudotime trajectory was defined using Monocle3. Subsequently, we analyzed *Prox1* expression and hepatocyte identity scores across this trajectory.

### Survival analysis

For all survival analyses, including patients and mouse models, survival durations were plotted as Kaplan–Meier curves, and a log-rank test was used to analyze the statistical significance of differences in survival outcomes. For patients with HCC survival impact analysis in Fig. 1e, −log_10_(*P*) from the log-rank test are shown, with values positive if survival was improved with higher candidate expression and negative if survival was poorer.

### Plasmid constructs

DNA constructs were generated by DNA synthesis (Sigma) or PCR amplification of cDNA with Q5 polymerase followed by ligation into restriction-digested vectors using indicated enzymes and T4 DNA ligase (NEB). All constructs and primers generated in this study can be found in Supplementary Tables 10 and 12, respectively.

### qPCR primers

DNA oligonucleotide primers for quantitative PCR were ordered from Sigma. All primers used in this study are described in Supplementary Table 12.

### Antibodies

All primary antibodies used in this study can be found in Supplementary Table 11. Secondary Alexa-conjugated antibodies for immunofluorescence were used at 1:2,000 (Invitrogen), and secondary IRDye-conjugated antibodies for western blot were used at 1:10,000 (LI-COR).

### Statistics and reproducibility

No statistical methods were used to predetermine the sample size for the experiments. Animals for primary cultures and in vivo experiments were selected randomly before indicated treatments. The investigators were blinded to the microscopy analysis and quantification. Otherwise, no blinding and randomization were performed. Figures 3a and 7a and ED Figs. 3a,e,m, 6c and 10a,g were created with BioRender.com and Affinity. Microsoft Excel v16.0 was used to create and organize Supplementary Tables 1–13.

### Reporting summary

Further information on research design is available in the Nature Portfolio Reporting Summary linked to this article.

## Online content

Any methods, additional references, Nature Portfolio reporting summaries, source data, extended data, supplementary information, acknowledgements, peer review information; details of author contributions and competing interests; and statements of data and code availability are available at 10.1038/s41588-025-02081-w.

## Supplementary information

Supplementary Information Supplementary Figs. 1–5.

Reporting Summary

Supplementary Tables Supplementary Tables 1–13.

Supplementary Data Statistical data for Supplementary Figs. 1, 3a–c, e, f and 4a.

## Source data

Source Data Fig. 6 and Extended Data Figs. 1–3, 5 and 8 Unprocessed western blots and gels.

Source Data Figs. 1–7 and Extended Data Figs. 1–5 and 8–10 Statistical source data.

## Data Availability

All data are present in the manuscript and the Supplementary Information. Raw mass spectrometry data have been deposited to the ProteomeXchange Consortium via the PRIDE partner repository (https://www.ebi.ac.uk/pride/login) under the dataset identifier PXD053043. Raw next-generation sequencing data can be found on Gene Expression Omnibus at accession GSE224832. A searchable database of our safeguard repressor analysis is available at https://apps.embl.de/safeguard/. Source data are provided with this paper.

## Code availability

The code used in this study is available at https://git.embl.de/grp-zaugg/safeguard-prox1 (ref. ^105^).
