# Supplementary material for: Active repression of cell fate plasticity by PROX1 safeguards hepatocyte identity and prevents liver tumorigenesis
Source: Nat Genet. 2025 Feb 13;57(3):668–79. doi: 10.1038/s41588-025-02081-w (PMC11906372; doi:10.1038/s41588-025-02081-w)
Supplement: Supplementary file 1 — Supplementary Figs. 1–5. [file 41588_2025_2081_MOESM1_ESM.pdf]

nature genetics

Supplementary information

https://doi.org/10.1038/s41588-025-02081-w

Active repression of cell fate plasticity by PROX1 safeguards hepatocyte identity and prevents liver tumorigenesis

In the format provided by the authors and unedited

Supplementary Information

Supplementary Figures 1 – 5

Supplementary Fig. 1: PROX1 chromatin binding sites in different tissues.

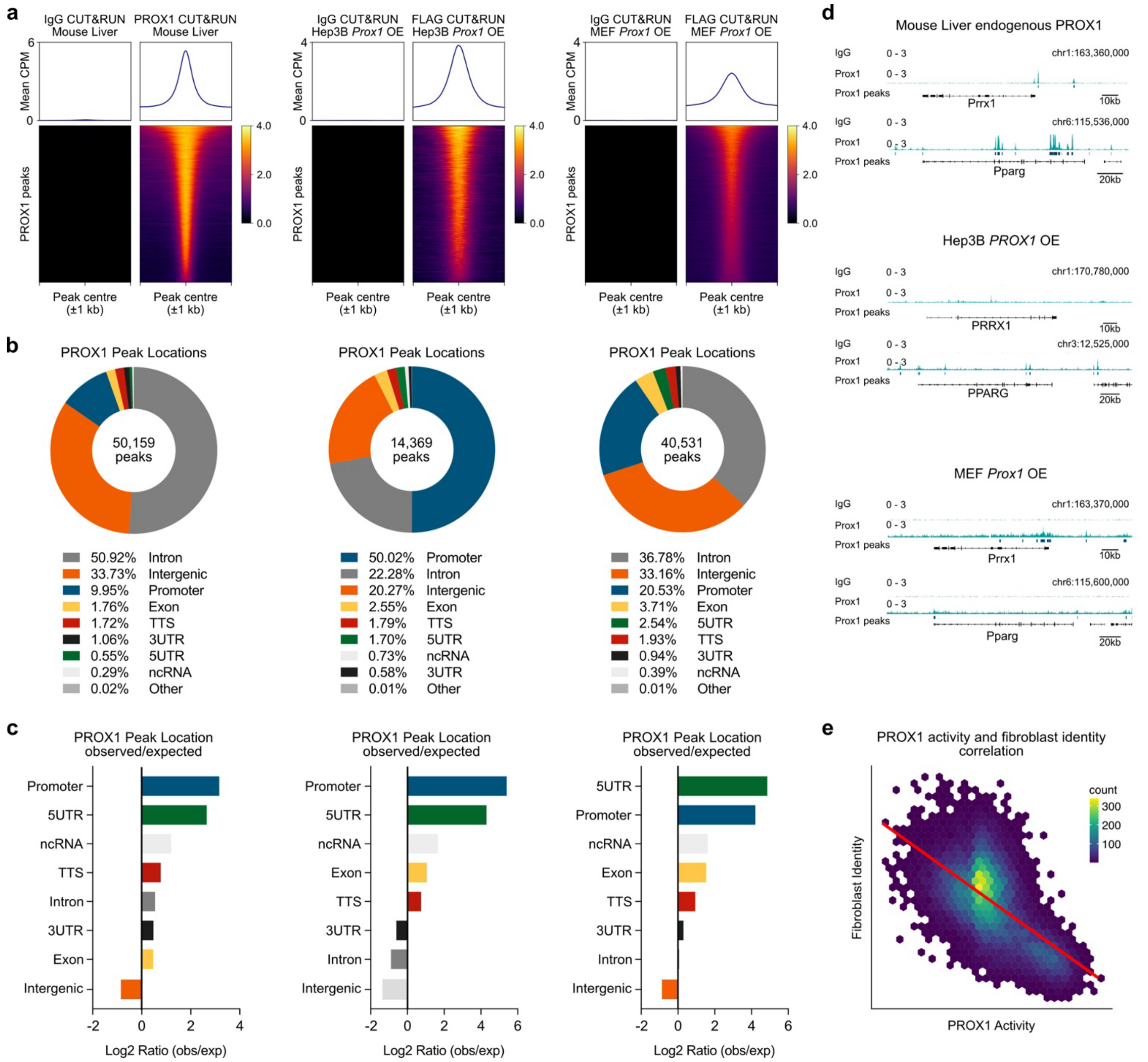

a, PROX1 chromatin binding was determined by CUT&RUN using PROX1 antibodies in primary mouse liver (n=5) or using FLAG antibodies upon overexpression of FLAG-tagged PROX1 in Hep3B (n=3) or MEFs (n=3) identifying high-confidence PROX1 binding sites compared to IgG. Heatmaps show PROX1 reads ±1 kb of the peak center. **b**, Proportion of PROX1 binding sites located in indicated genomic regions from experiments in (**a**). **c**, Log2 ratio of observed PROX1 binding sites from (**a**) to expected binding highlight enrichment at promoters. **d**, Genome browser tracks showing example regions of PROX1 binding in (**a**) at selected non-hepatocyte genes. **e**, Negative correlation of fibroblast identity score with PROX1 activity across all single cells during hepatocyte reprogramming time course. p-value < 2.2e-16.

Supplementary Fig. 2: Single-cell transcriptome analysis during hepatocyte reprogramming time course.

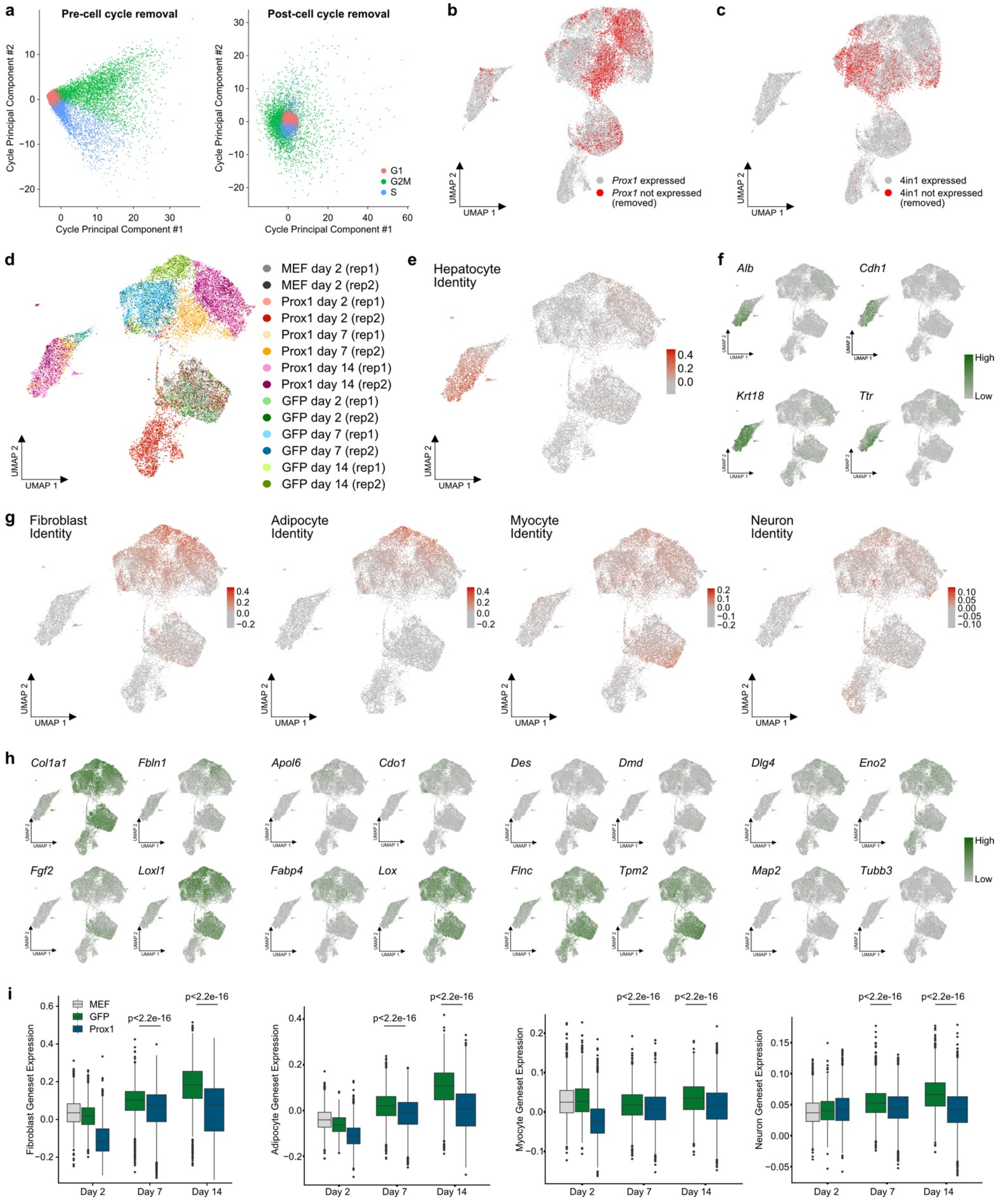

a, PCA of cells based on cell cycle gene expression pre- and post-adjustment for cell cycle effects with Seurat. b,**c**, UMAP of cells labeled by expression of *Prox1* (**b**) and 4in1 (**c**), inferred from PROX1 and 4in1 activity. **d**, UMAP of 22,761 cells labeled by condition and replicate following demultiplexing using barcode oligos. **e**, Hepatocyte identity score in each cell on UMAP. **f**, Expression of selected individual hepatocyte marker genes in all cells. **g**, Fibroblast, adipocyte, myocyte, and neuron identity scores projected onto the UMAP. **h**, Expression of selected fibroblast, adipocyte, myocyte, and neuronal marker genes in all cells. **i**, Quantification of fibroblast, adipocyte, myocyte, and neuron identity scores across all cells. Boxplots show median and interquartile range (IQR), whiskers = 1.5xIQR, n=2. Unpaired two-tailed t-test, p-values displayed.

Supplementary Fig. 3: PROX1 inhibits reprogramming to myocyte and neuronal cell fates.

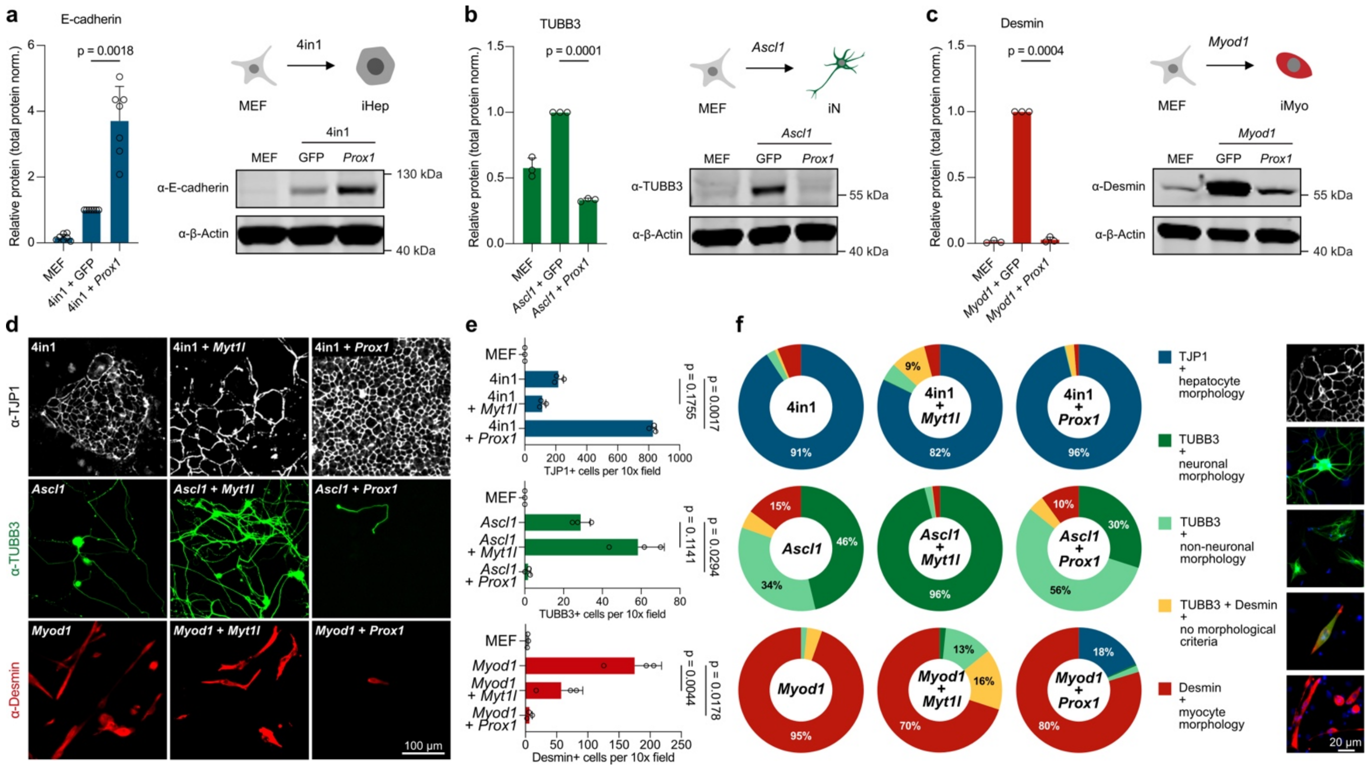

a-c, Reprogramming of MEFs to induced hepatocytes, neurons, or myocytes using 4in1, *Ascl1*, or *Myod1* overexpression, respectively. Protein quantification of E-cadherin (hepatocyte), TUBB3 (neuronal), or Desmin (myocyte) marker proteins at day 14 of respective reprogramming protocols with or without *Prox1* overexpression using Western blot analysis. **d**, Representative immunofluorescence microscopy of cells reprogrammed as in (**a-c**) with or without *Prox1* or *Myt1l* overexpression in respective reprogramming protocols using indicated antibodies (n=3). **e**, Quantification of successfully reprogrammed cells in (**d**), based on displayed immunofluorescence markers and morphology. **f**, Percentage of reprogrammed cells in (**d**) positive for indicated markers and morphological criteria. Scale bar 100 µm and 20 µm (**d, f**). Bar graphs show mean from n=7 (**a**) or n=3 (**b, c, e**), error bars = SD, two-tailed Dunnett's test, p-values displayed.

Supplementary Fig. 4: PROX1 target gene repression mimics PROX1 function.

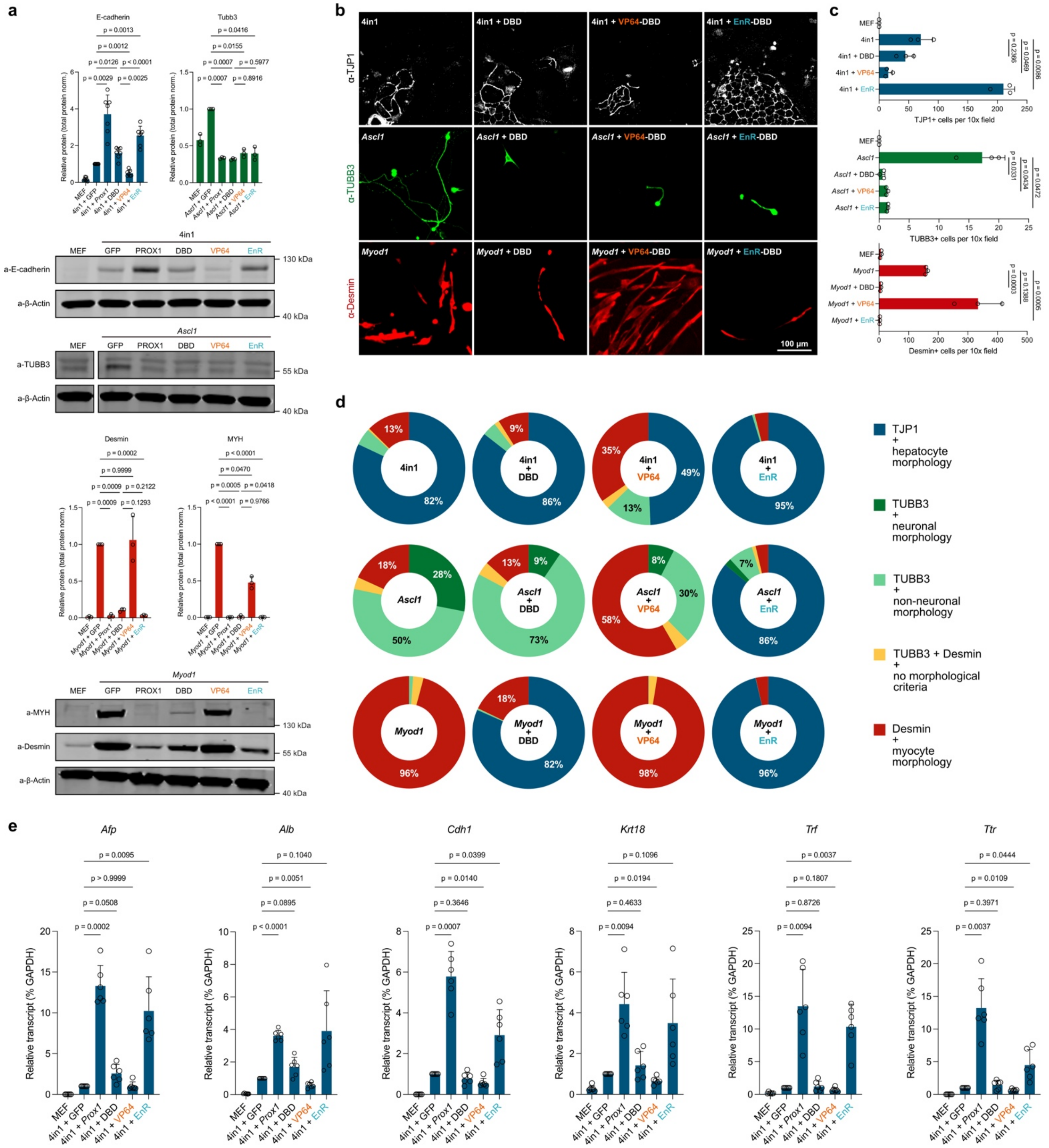

a, Reprogramming of MEFs to induced hepatocytes, neurons, or myocytes using *MyoD1*, *Ascl1*, or 4in1 overexpression, respectively. PROX1 target gene activation was induced by fusion of PROX1 DNA-binding domain (DBD) to the VP64 activator, while silencing was triggered by EnR repressor fusion. Full-length PROX1 and DBD served as controls. Protein quantification of E-cadherin (hepatocyte, n=7), Tubb3 (neuronal, n=3), or MYH and Desmin (muscle, n=3) marker proteins at day 7 of each reprogramming experiment using Western blot analysis. **b**, Immunofluorescence microscopy images of cells in (**a**) at day 14 of reprogramming using indicated antibodies (n=3). **c**, Quantification of reprogrammed cells in (**b**) based on displayed immunofluorescence markers and morphology. **d**, Percentage of reprogrammed cells in (**b**) positive for indicated markers and morphological criteria. **e**, Expression of indicated hepatocyte marker genes at day 7 of 4in1-induced hepatocyte reprogramming upon overexpression of indicated PROX1 fusion constructs based on qRT-PCR (n=6). Scale bar 100 µm (**b**). Bar graphs show mean from specified number of replicates, error bars = SD, two-tailed Dunnett's test, p-values displayed.

Supplementary Fig. 5: Induction of hepatocyte genes and repression of alternate fates by PROX1.

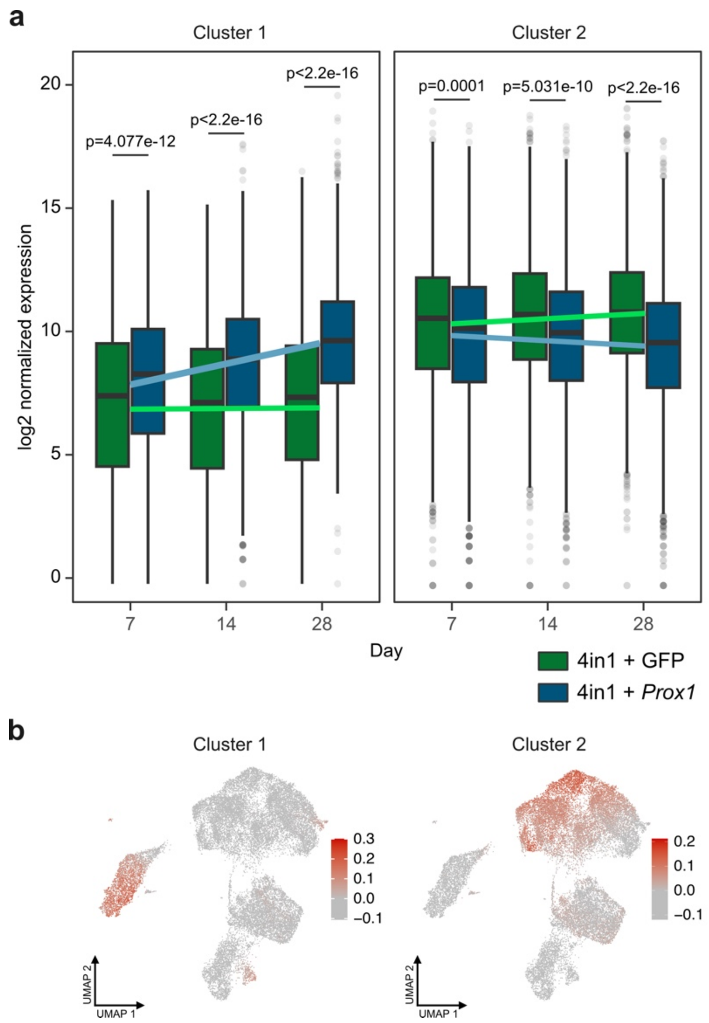

a, Bulk gene expression changes during 4in1-induced hepatocyte reprogramming at indicated time points clustered by differential expression and chromatin accessibility. Median gene expression of 4,185 differentially expressed genes between Prox1 and GFP control are presented as boxplots for both clusters (n=2). **b**, Aggregate expression of genes from bulk gene expression-derived clusters 1 and 2 projected onto UMAP of 4in1-induced reprogramming single-cell transcriptomics data, highlighting overlap of cluster 1 with hepatocyte cluster and cluster 2 with alternate fate cluster (n=2). Boxplots show median and interquartile range (IQR), whiskers = 1.5xIQR, n=2. Unpaired two-tailed t-test, p-values displayed.
