# Supplementary material for: Active repression of cell fate plasticity by PROX1 safeguards hepatocyte identity and prevents liver tumorigenesis
Source: Nat Genet. 2025 Feb 13;57(3):668–79. doi: 10.1038/s41588-025-02081-w (PMC11906372; doi:10.1038/s41588-025-02081-w)
Supplement: Supplementary file 2 — Reporting Summary [file 41588_2025_2081_MOESM2_ESM.pdf]

nature portfolio

Corresponding author(s): Moritz Mall
Judith B Zaugg

Last updated by author(s): Dec 17, 2024

Reporting Summary

Nature Portfolio wishes to improve the reproducibility of the work that we publish. This form provides structure for consistency and transparency in reporting. For further information on Nature Portfolio policies, see our Editorial Policies and the Editorial Policy Checklist.

Statistics

For all statistical analyses, confirm that the following items are present in the figure legend, table legend, main text, or Methods section.

n/a	Confirmed	
☐	☒	The exact sample size (*n*) for each experimental group/condition, given as a discrete number and unit of measurement
☐	☒	A statement on whether measurements were taken from distinct samples or whether the same sample was measured repeatedly
☐	☒	The statistical test(s) used AND whether they are one- or two-sided<br>*Only common tests should be described solely by name; describe more complex techniques in the Methods section.*
☐	☒	A description of all covariates tested
☐	☒	A description of any assumptions or corrections, such as tests of normality and adjustment for multiple comparisons
☐	☒	A full description of the statistical parameters including central tendency (e.g. means) or other basic estimates (e.g. regression coefficient) AND variation (e.g. standard deviation) or associated estimates of uncertainty (e.g. confidence intervals)
☐	☒	For null hypothesis testing, the test statistic (e.g. *F*, *t*, *r*) with confidence intervals, effect sizes, degrees of freedom and *P* value noted<br>*Give P values as exact values whenever suitable.*
☒	☐	For Bayesian analysis, information on the choice of priors and Markov chain Monte Carlo settings
☒	☐	For hierarchical and complex designs, identification of the appropriate level for tests and full reporting of outcomes
☐	☒	Estimates of effect sizes (e.g. Cohen's *d*, Pearson's *r*), indicating how they were calculated

Our web collection on statistics for biologists contains articles on many of the points above.

Software and code

Policy information about availability of computer code

Data collection	R v4.2.0 and Microsoft Excel v16.0 were used in collating and cleaning data for analysis.
Data analysis	Aperio ImageScope (v12.4.0.5043, Leica) and Fiji (ImageJ v1.53q) for histology.<br>Data analysis for small n (n<10) was performed in GraphPad Prism v9.0.<br>For bulk RNA-seq, reads were mapped using STAR and differential expression was determined using DESeq2 (R package version 1.28.1).<br>CUT&RUN data were analysed with nf-core/cutandrun pipeline v1.0.0.<br>For ATAC-seq, reads were quality-checked with fastqc (v0.11.8), trimmed with trimmomatic (v0.38), aligned to UCSC mm10 with bowtie2 (v2.3.4.3, v2.4.2), then cleaned and base-recalibrated (to take account of Tn5 insertion biases) with samtools (v1.10) and picard (v2.18.16). Reads were filtered with bedtools (v2.27.1, v2.30.0), samtools, and picard. Peaks were called using Genrich (v0.6.1) and coverage was calculated with deeptools (v3.1.3, v3.5.0). Final quality checks were performed with multiqc (v1.6, v1.11). Differential peak analysis was performed with DiffBind (v3.4.11).<br>scRNA-seq data were analysed with Seurat (v4.0, v4.3).<br>Alignments were performed to hg38 for human cells or mm10 for mouse.<br>Biorender and Affinity were used for figure generation.

For manuscripts utilizing custom algorithms or software that are central to the research but not yet described in published literature, software must be made available to editors and reviewers. We strongly encourage code deposition in a community repository (e.g. GitHub). See the Nature Portfolio guidelines for submitting code & software for further information.

Data

Policy information about availability of data

All manuscripts must include a data availability statement. This statement should provide the following information, where applicable:

- Accession codes, unique identifiers, or web links for publicly available datasets
- A description of any restrictions on data availability
- For clinical datasets or third party data, please ensure that the statement adheres to our policy

All data are present in the manuscript and the supplementary materials. Raw mass spectrometry data have been deposited to the ProteomeXchange Consortium via the PRIDE partner repository (https://www.ebi.ac.uk/pride/login) under the dataset identifier PXD053043. Raw next-generation sequencing data can be found on GEO at accession GSE224832. A searchable database of our safeguard repressor analysis is available at: apps.embl.de/safeguard.

Research involving human participants, their data, or biological material

Policy information about studies with human participants or human data. See also policy information about sex, gender (identity/presentation), and sexual orientation and race, ethnicity and racism.

Reporting on sex and gender	Sex and gender reporting was performed for human data.
Reporting on race, ethnicity, or other socially relevant groupings	Race/ethnicity reporting was not performed for human data.
Population characteristics	Samples were collected from patients with primary liver cancer in Mannheim, Germany. Covariate-relevant population characteristics, specifically age and gender, were included in the raw data tables provided as part of the Source Data.
Recruitment	Human liver tissue samples were collected from patients with primary liver cancer at the Medical Faculty Mannheim, Heidelberg University.
Ethics oversight	The study was approved by the Institutional Review Board (IRB) of the University Hospital Mannheim, Medical Faculty Mannheim of the University of Heidelberg (Theodor-Kutzer-Ufer 1-3, 68167 Mannheim, Germany) under permit number 2012-293N-MA. Specimens were collected with informed patient consent in accordance with this IRB approval.

Note that full information on the approval of the study protocol must also be provided in the manuscript.

Field-specific reporting

Please select the one below that is the best fit for your research. If you are not sure, read the appropriate sections before making your selection.

☒ Life sciences ☐ Behavioural & social sciences ☐ Ecological, evolutionary & environmental sciences

For a reference copy of the document with all sections, see nature.com/documents/nr-reporting-summary-flat.pdf

Life sciences study design

All studies must disclose on these points even when the disclosure is negative.

Sample size	No statistical methods were used to predetermine the sample size for the experiments. Sample sizes were determined based on prior experiments and established standards in the field, including Moritz Mall et al., Nature, 2017 for reprogramming; Darjus F. Tschaharganeh et al., Cell, 2014 for cancer; and Lu Li et al., Cell Stem Cell, 2023 for injury.
Data exclusions	No data were excluded.
Replication	The experiments were performed in three or more independent biological replicates, as the figure legends indicate. One exception is one cell line experiment (Myc/Trp53) that was performed in two biological replicates with 6 technical replicates each. Wherever applicable, attempts at reproducibility were successful.
Randomization	Allocation of samples and animals to experimental groups was random. Primary cultures were randomly prepared and mice were randomly treated.
Blinding	Animals for primary cultures and in vivo experiments were randomly selected before the indicated treatments. Investigators were blinded during microscopy analysis and quantification to minimize bias. Otherwise, no additional blinding or randomization was performed. This approach follows standard practices in the field and allowed the experiments to be conducted efficiently with a limited number of researchers.

Reporting for specific materials, systems and methods

We require information from authors about some types of materials, experimental systems and methods used in many studies. Here, indicate whether each material, system or method listed is relevant to your study. If you are not sure if a list item applies to your research, read the appropriate section before selecting a response.

Materials & experimental systems

n/a	Involved in the study
☐	☒ Antibodies
☐	☒ Eukaryotic cell lines
☒	☐ Palaeontology and archaeology
☐	☒ Animals and other organisms
☒	☐ Clinical data
☒	☐ Dual use research of concern
☒	☐ Plants

Methods

n/a	Involved in the study
☒	☐ ChIP-seq
☒	☐ Flow cytometry
☒	☐ MRI-based neuroimaging

Antibodies

Antibodies used

Primary antibodies: A6 (rat) from Hybridoma Bank, dilution 1:50 (IHC); Alb (goat) from Bethyl (A90-134A), dilution 1:1,000 (WB); B220 (rat) from BD Biosciences (553084), dilution 1:3,000 (IHC); β-Actin (mouse) from Sigma-Aldrich (A5441), dilution 1:10,000 (WB); Casp3 (rabbit) from Cell Signaling Technology (9661), dilution 1:300 (IHC); CD3 (rabbit) from Invitrogen (MA1-90582), dilution 1:500 (IHC); CD8 (rat) from Invitrogen (14-0808-82), dilution 1:200 (IHC); CK19 (rat) from Hybridoma Bank (Antibody Registry ID: AB_2133570), dilution 1:500 (IHC). Clec4F (goat) from R&D (AF2784-SP), dilution 1:1,000 (IHC); Desmin (rabbit) from Abcam (ab32362), dilution 1:500 (ICC), 1:1,000 (WB); Ecadh (rabbit) from Cell Signaling Technology (24E10), dilution 1:1,000 (WB); F4/80 (rat) from Linaris (T2006), dilution 1:250 (IHC); FLAG M2 (mouse) from Sigma-Aldrich (F1804), dilution 1:1,000 (ICC, WB); FLAG (rabbit) from Cell Signaling 14793 (C&R); GFP (rabbit) from Invitrogen (A11122), dilution 1:250 (IHC); HNF4 (rabbit) from Abcam (ab181604), dilution 1:2,000 (IHC); Ki-67 (rabbit) from Thermo Scientific (RM-9106-S1), dilution 1:200 (IHC); PROX1 (rabbit) from ReliaTech (RLT-102-PA32S), dilution 1:500 (WB); Sox9 (rabbit) from Abcam (ab185230), dilution 1:2,000 (IHC); TJP1 (rat) from Invitrogen (14-9776-80), dilution 1:500 (ICC); TUBB3 (mouse) from BioLegend (801202), dilution 1:1,000 (ICC, WB).

Secondary antibodies: Alexa Fluor 488 Goat (anti-guinea pig) from Thermo Fisher Scientific (Carlsbad, USA), dilution 1:2,000 (ICC); Alexa Fluor 488 Donkey (anti-mouse) from Thermo Fisher Scientific (Carlsbad, USA), dilution 1:2,000 (ICC); Alexa Fluor 555 Donkey (anti-rabbit) from Thermo Fisher Scientific (Carlsbad, USA), dilution 1:2,000 (ICC); Alexa Fluor 647 Donkey (anti-rat) from Thermo Fisher Scientific (Carlsbad, USA), dilution 1:2,000 (ICC); IRDye 680RD Donkey (anti-mouse) from LI-COR Biosciences (Lincoln, USA), dilution 1:10,000 (WB); IRDye 800CW Donkey (anti-rabbit) from LI-COR Biosciences (Lincoln, USA), dilution 1:10,000 (WB).

Validation

All antibodies used in this study were commercially obtained. Validation was performed using positive and negative control samples for the respective applications, either by the provider or by us.

Eukaryotic cell lines

Policy information about cell lines and Sex and Gender in Research

Cell line source(s)

MEF lines were obtained as primary cultures from E13.5 mouse embryos. The distal portions of all limbs from 3-4 embryos were dissected, placed in 100 µL trypsin, cut thoroughly, and incubated in a total of 1 mL trypsin (37°C, 15 min). Trypsin was inactivated by the addition of cell suspension to 25 mL MEF media (DMEM; Invitrogen) containing 10% cosmic calf serum (CCS; Hyclone), beta-mercaptoethanol (Sigma), non-essential amino acids, sodium pyruvate, L-glutamine, and penicillin/streptomycin (all from Invitrogen). MEFs were then cultured in MEF media and either cryopreserved or passaged twice using Trypsin before reprogramming experiments.

Hep3B human cell lines were obtained from ATCC (HB-8064).
HEK-293T cell lines were sourced from ATCC (CRL-1573).

Authentication

Cell lines were not authenticated.

Mycoplasma contamination

All cell lines were tested regularly for mycoplasma contamination and were found negative.

Commonly misidentified lines
(See ICLAC register)

No commonly misidentified cell lines were used in this study.

Animals and other research organisms

Policy information about studies involving animals; ARRIVE guidelines recommended for reporting animal research, and Sex and Gender in Research

Laboratory animals

Mus musculus C57BL/6N 12 weeks (HDTV) or C57BL/6J (Prox1fl/fl) and C57BL/6N pregnant with E13.5 embryos (MEF harvest).

Wild animals	No wild animals were used in this study.
Reporting on sex	Animal experiment data for tail vein injection were generated from female mice only and injury model was performed in male mice only. Sex was considered only with regards to technical aspects of tail vein injection (which is more complex with male mice) and nd mouse availability (male Prox1 floxed animals). For all cell line-based experiments, sex was not considered as a factor.
Field-collected samples	No field-collected samples were used.
Ethics oversight	All animal experiments were approved by the regional ethics board, Karlsruhe, Germany, and performed in accordance with their regulations.

Note that full information on the approval of the study protocol must also be provided in the manuscript.

Plants

Seed stocks	N/A
Novel plant genotypes	N/A
Authentication	N/A
