# Supplementary figures and images for: Active repression of cell fate plasticity by PROX1 safeguards hepatocyte identity and prevents liver tumorigenesis
Source: Nat Genet. 2025 Feb 13;57(3):668–79. doi: 10.1038/s41588-025-02081-w (PMC11906372; doi:10.1038/s41588-025-02081-w)

Ext. Fig. 11

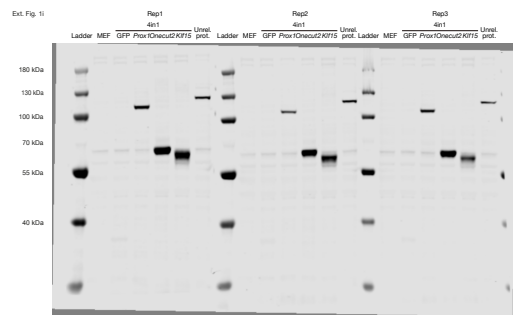

Ext. Fig. 25

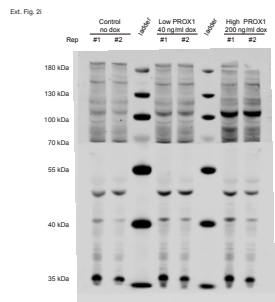

Sup. Fig. 3a

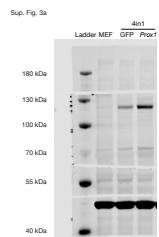

Sup. Fig. 3b

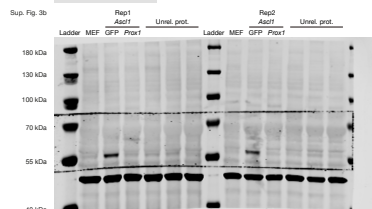

Sup. Fig. 3c

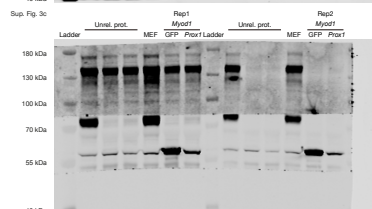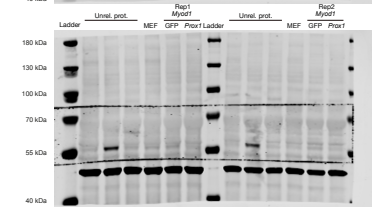

Ext. Fig. 1j

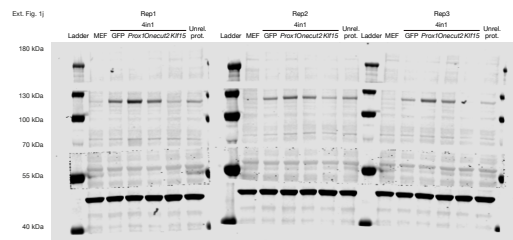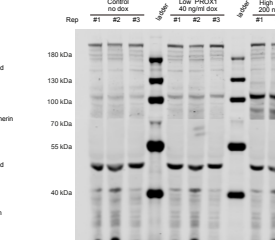

Ext. File #

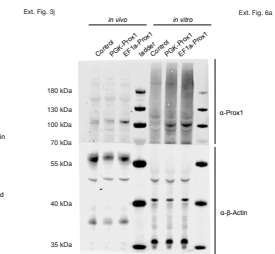

Ext. Fig. 6a

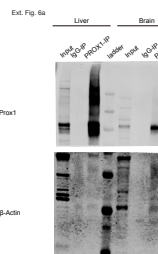

Fig. 6g  
Ext. Fig. 9b

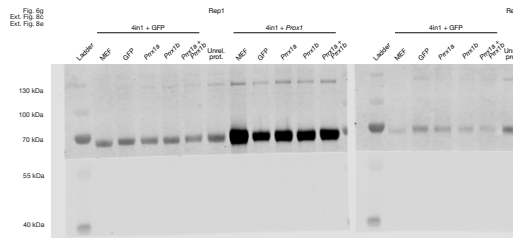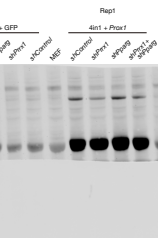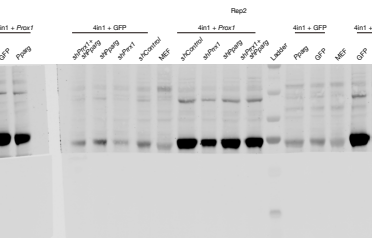

Ext. Fig. 5a

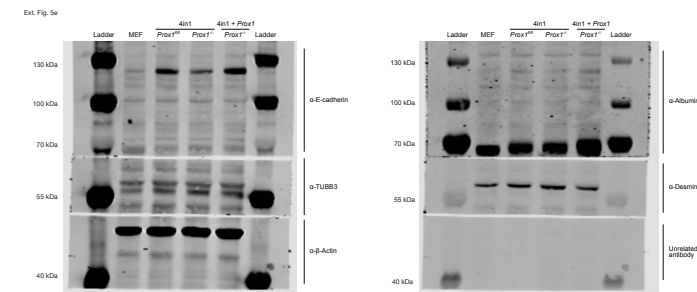

Sup. Fig. 4a

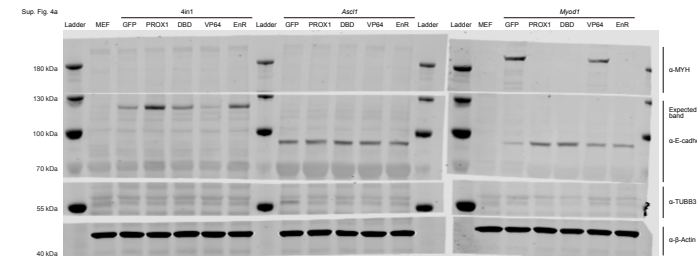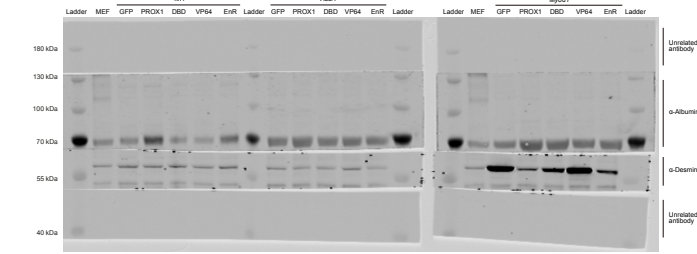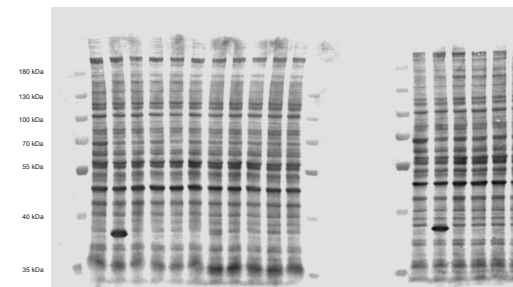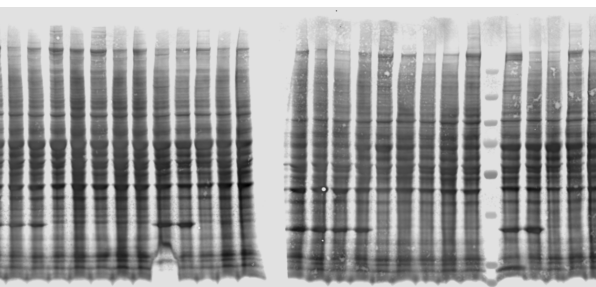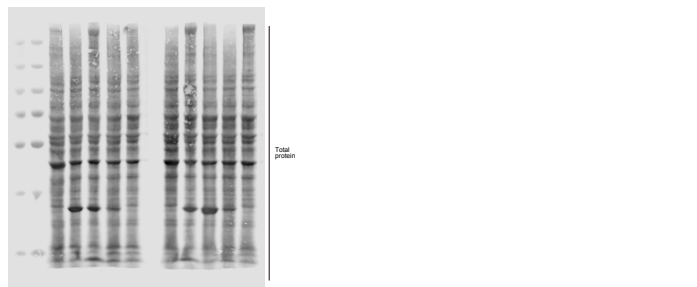

Supplement: Supplementary file 5 — Unprocessed western blots and gels. [file 41588_2025_2081_MOESM5_ESM.pdf]
